# An ancestral haplotype of the human *PERIOD2* gene associates with reduced sensitivity to light-induced melatonin suppression

**DOI:** 10.1371/journal.pone.0178373

**Published:** 2017-06-26

**Authors:** Tokiho Akiyama, Takafumi Katsumura, Shigeki Nakagome, Sang-il Lee, Keiichiro Joh, Hidenobu Soejima, Kazuma Fujimoto, Ryosuke Kimura, Hajime Ishida, Tsunehiko Hanihara, Akira Yasukouchi, Yoko Satta, Shigekazu Higuchi, Hiroki Oota

**Affiliations:** 1 Department of Anatomy, Kitasato University School of Medicine, Sagamihara, Kanagawa, Japan; 2 Department of Biosciences, School of Science, Kitasato University, Sagamihara, Kanagawa, Japan; 3 Department of Evolutionary Studies of Biosystems, SOKENDAI (The Graduate University for Advanced Studies), Hayama, Kanagawa, Japan; 4 Department of Mathematical Analysis and Statistical Inference, The Institute of Statistical Mathematics, Tachikawa, Tokyo, Japan; 5 Department of Human Genetics, University of Chicago, Chicago, Illinois, United States of America; 6 Department of Human Science, Faculty of Design, Kyushu University, Minami-ku Fukuoka, Japan; 7 Division of Molecular Genetics and Epigenetics, Department of Biomolecular Science, Faculty of Medicine, Saga University, Nabeshima, Saga, Japan; 8 Department of Internal Medicine, Faculty of Medicine, Saga University, Nabeshima, Saga, Japan; 9 Department of Human Biology and Anatomy, Faculty of Medicine, University of the Ryukyus, Nishihara-cho, Okinawa, Japan; 10 Department of Biological Structure, Kitasato University Graduate School of Medical Sciences, Sagamihara, Kanagawa, Japan; University of Lübeck, GERMANY

## Abstract

Humans show various responses to the environmental stimulus in individual levels as “physiological variations.” However, it has been unclear if these are caused by genetic variations. In this study, we examined the association between the physiological variation of response to light-stimulus and genetic polymorphisms. We collected physiological data from 43 subjects, including light-induced melatonin suppression, and performed haplotype analyses on the clock genes, *PER2* and *PER3*, exhibiting geographical differentiation of allele frequencies. Among the haplotypes of *PER3*, no significant difference in light sensitivity was found. However, three common haplotypes of *PER2* accounted for more than 96% of the chromosomes in subjects, and 1 of those 3 had a significantly low-sensitive response to light-stimulus (*P* < 0.05). The homozygote of the low-sensitive *PER2* haplotype showed significantly lower percentages of melatonin suppression (*P* < 0.05), and the heterozygotes of the haplotypes varied their ratios, indicating that the physiological variation for light-sensitivity is evidently related to the *PER2* polymorphism. Compared with global haplotype frequencies, the haplotype with a low-sensitive response was more frequent in Africans than in non-Africans, and came to the root in the phylogenetic tree, suggesting that the low light-sensitive haplotype is the ancestral type, whereas the other haplotypes with high sensitivity to light are the derived types. Hence, we speculate that the high light-sensitive haplotypes have spread throughout the world after the Out-of-Africa migration of modern humans.

## Introduction

Circadian rhythms, as endogenous self-sustained oscillations of a period of approximately 24 hours, are synchronized with a 24-hour cycle of the external environment by the circadian clock system which accesses light-dark information [1,2]. The light information received by the retina is carried through the retinohypothalamic tract to the brain, and causes various biological reactions in humans, for instance, phase response of circadian rhythms [3], alerting effects of light [4], pupillary reflex [5], suppression of melatonin secretion [6], and cognitive brain function [7]. These reactions caused by light are termed “non-image-forming” responses [8].

Typically the circadian light sensitivity is quantified by the measurements of melatonin secretion. Regulated by the circadian clock, melatonin is secreted from the pineal gland at night. It is acutely suppressed by light exposure during nighttime, so the suppression of melatonin secretion is used as an index for the circadian light sensitivity [9]. Suppression of melatonin was first demonstrated by exposure to bright light at 2500 lx in humans [6]. A recent study has shown that melatonin secretion is suppressed by a lower illuminance level (<200 lx) of room light [10]. There is individual variation of light sensitivity based on melatonin suppression by light [11]. It has been shown that factors causing the variation in melatonin suppression include light exposure history in daytime [12], seasonal differences [13], differences of eye color and ethnicity [14], and age differences [15]. However, genetic factors causing these variations are still not well known; as well as any other physiological variations in humans, it has been thought that the variation of light sensitivity based on melatonin suppression is attributed largely to a plastic change without genetic programming [16].

It is reported that variations in clock genes (e.g., *PERIOD2*, *CLOCK*, and *casein kinase 1 epsilon* [*CK1ε*]) are related to disorders in sleep or circadian rhythmicity in humans [17,18]. Regarding *PERIOD* (*PER*) genes that are the central factors of the transcriptional regulation network of clock genes [19,20], their variations have been studied from the perspective of evolution [21]. Differences of allele frequency in the *PER2* gene have been reported among worldwide populations [22]. A variable number tandem repeat (VNTR) polymorphism in the *PER3* gene exhibiting different allelic frequencies has also been reported among worldwide populations [23]. These results imply geographical differentiation of the *PER2* and *PER3* genes in human populations. A previous study argues that in six clock genes, including *PER2* and *PER3*, the allele frequency differences among worldwide populations are likely to be caused by genetic drift rather than natural selection [24]. Meanwhile, in the case of the *period* gene of other animals, for example, *Drosophila melanogaster*, allele frequencies of different Thr-Gly length variants show a latitudinal cline [25], and it is argued that the polymorphism is maintained through selection [26,27]. Therefore, it is of interest whether or not variations of *PER2* and *PER3* genes are related to adaptation in geographical circumstances.

However, to our knowledge, what physiological variations could be associated with the cause of the geographical differentiation of allele frequencies in *PER* genes in humans has never been examined. Therefore, we hypothesize that the *PER* genes harbored polymorphisms that would explain physiological variations related to circadian light sensitivity. To clarify this hypothetical issue on the *PER* genes, we collected three major types of physiological data from 43 healthy subjects, and performed genotyping on them for the *PER2* and *PER3* genes. We also genotyped 91 non-subject volunteers, and then compared the resultant data with that from 850 individuals from 10 worldwide populations by means of the 1000 Genome Project database [28]. Here, we examine the association between the *PER2* haplotypes and the percentages of melatonin suppression and touch on their evolutionary history in modern humans.

## Materials and methods

### Measurements of physiological data

As subjects of our physiological experiments, a total of 43 healthy 18- to 24-year-old students (21 males and 22 females) in Kyushu University (Fukuoka, Japan) participated and were assayed in a series of 3-set tests for physiological responses to light stimulus. The experiment was performed from January through March in 2011. Each subject completed a Japanese version of the Morningness-Eveningness Questionnaire (MEQ) [29,30]. One week prior to the start of the experiment, the subjects were instructed to keep their habitual sleep-wake rhythms by conducting self-recording of the sleep diary (bedtime, rising time, sleep quality, etc.). The average ± standard deviation (SD) of bedtime, wake time, and midpoint of sleep was 1:44 ± 0:59, 9:31 ± 1:16, 5:38 ± 1:00, respectively.

To assess the percentages of melatonin suppression and those of pupil constriction, the subjects were kept under dim light for 4 hours and then exposed to bright light for 3 hours. The illuminance level of the dim light was set at or below 15 lx. As a light source white fluorescent light from the ceiling (5000 K) was used. Its illuminance level in the vertical direction was 1000 lx, measured at the subjects’ eye level using a light meter (CL-200, Konica Minolta Holdings, Inc., Tokyo, Japan). During the exposure to light, each subject sat on a chair and watched an unexciting movie to fix his or her eyes. A 7-inch liquid crystal display (LCD) was placed about 50 cm in front of the subject. The light level of the LCD was very low (<5 lx) compared to that of the room light. The vertical position of each subject’s head from the room light was adjusted to the same position to minimize the difference in light level due to the subject’s height. The experimental room was monitored by video camera to check the subjects’ eyes, posture, and angle of gaze, although the real-time measurements of light level of each subject during light exposure were not conducted.

The start time of light exposure was determined based on the individual midpoint of sleep, which is highly correlated with dim light melatonin onset (DLMO) [31]. We set the start time of light exposure between 3 and 3.5 hours before the time of sleep midpoint in the present study. According to the estimation in the previous study [31], this time zone corresponds approximately to 3 hours after DLMO. The average time of light exposure was 02:29 ± 0:58.

The subjects provided saliva samples every hour during these sessions. Salivette (Sarstedt AG & Co., Nümbrecht, Germany) was used for collecting samples of saliva. For the measurement of melatonin concentration in saliva, we conducted radioimmunoassays (RK-DSK, Buhlmann, Schonenbuch, Switzerland). In addition, we asked the subjects to wake up in middle of the night to collect a saliva sample as additional control data at home 2 days before the experiments began. The measurement time corresponded to 3 hours after light exposure on the experimental night.

The percentage of melatonin suppression was calculated based on the values before exposure to light as in the previous studies [13,14]. The percentage of suppression of melatonin concentration after light exposure was defined as [(melatonin concentration before exposure to light − melatonin concentration after exposure to light) / melatonin concentration before exposure to light] × 100. The percentages of melatonin suppression were also calculated based on the control sample that was taken at home in middle of the night. There was a significantly high correlation between the percentages of melatonin suppression based on the data before light exposure and those based on the data from the control sample taken at home (r = 0.725). In the present study, we used the data based on before light exposure because samples taken at home were missing from five subjects. For the following association studies, the percentage of melatonin suppression at 3 hours after light exposure was used as an index of circadian photosensitivity.

Steady-state pupil area of the right eye was measured under dim light condition and 1 hour after light exposure for 1 minute using an electronic infrared pupillometer (DK-101, Scalar Corporation, Tokyo, Japan). Average pupil size was calculated. The percentage of constriction of pupil size of light exposure was defined as [(pupil area before exposure to light − pupil area during exposure to light) / pupil area before exposure to light] × 100.

### DNA from the subjects

We extracted DNA from the saliva using phenol-chloroform extraction and the Gentra Puregene Blood Kit (Qiagen). The physiological experiments were conducted with written informed consent, and the ethics committees for human subjects at Kyushu University and at Kitasato University approved all the sampling protocol.

### Non-subject samples

To ensure that the selection of subjects was not biased and the following association studies using the subjects were not affected by subpopulation structures, we examined 91 controls: 51 were from Northern Kyushu (all of their grandparents were born in Saga, Nagasaki, or Fukuoka prefectures, confirmed by interview) and 40 were from the Ryukyu Islands (all of their grandparents were born in the Okinawa or Sakishima Islands, confirmed by interview). All the saliva samples were provided with written informed consent, and this research was approved by the ethics committees at Saga University, University of the Ryukyus, and Kitasato University. DNA samples from the Ryukyu individuals were prepared with the Oragene DNA Self-Collection Kit (DNA Genotek Inc., Ontario, Canada) (Nakagome et al. in preparation). For the other saliva samples of 51 individuals collected using the Oragene DNA Self-Collection Kit (DNA Genotek Inc.), DNA extraction was performed using the Gentra Puregene Blood Kit (Qiagen).

The genotype data of 10 worldwide human populations were extracted from the 1000 Genomes Project (phase 1) database [28]. They are two African populations (Luhya in Webuye, Kenya [LWK] N = 97, Yoruba in Ibadan, Nigeria [YRI] N = 88), five European populations (Utah residents with Northern and Western European ancestry [CEU] N = 85, Finnish in Finland [FIN] N = 93, British in England and Scotland [GBR] N = 89, Iberian populations in Spain [IBS] N = 14, Toscani in Italia [TSI] N = 98), two East Asian populations (Han Chinese in Beijing, China [CHB] N = 97, Southern Han Chinese, China [CHS] N = 100), and a Japanese population (Japanese in Tokyo, Japan [JPT] N = 89).

### SNP typing

**Fig 1:**
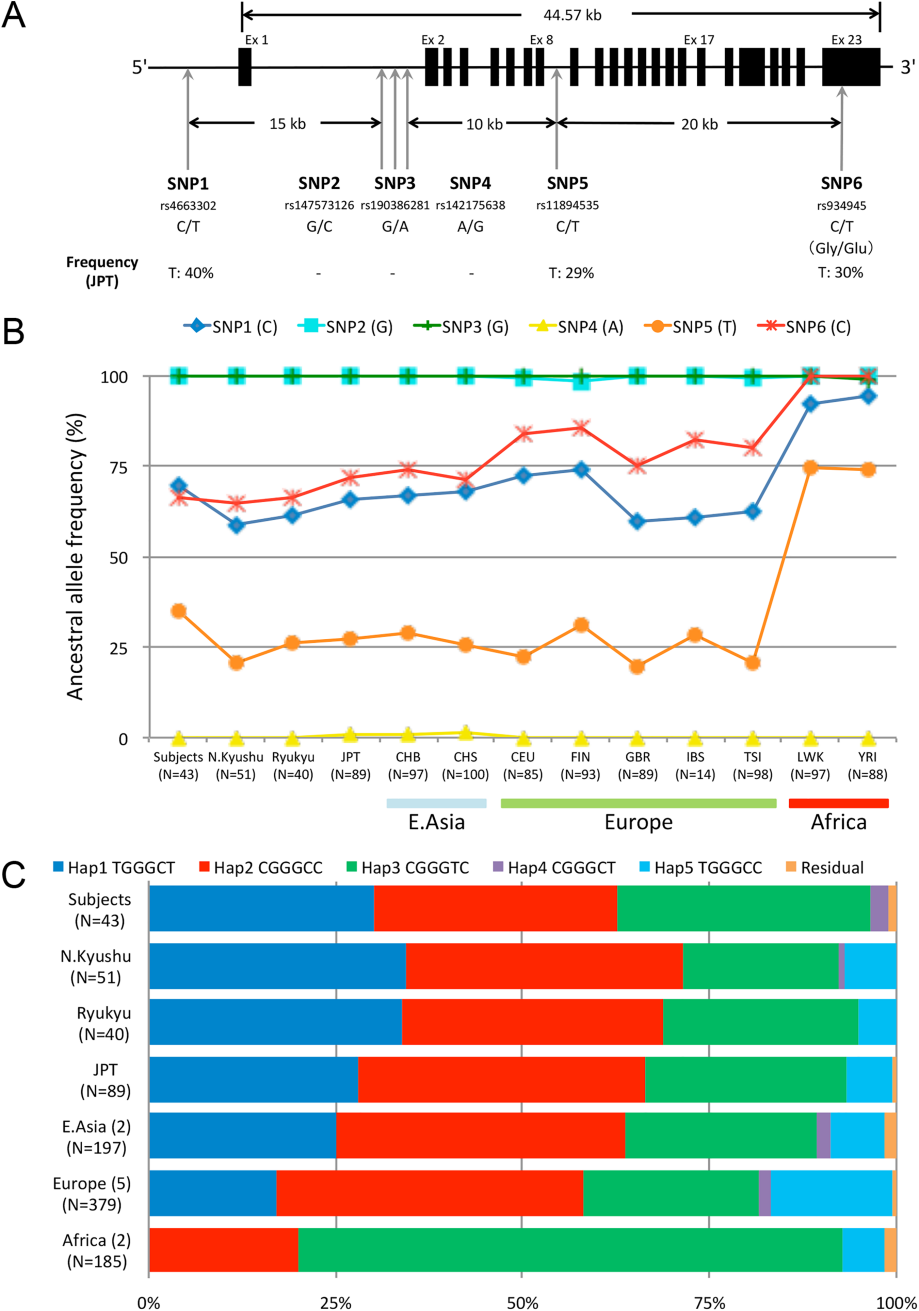
SNPs examined in the *PER2* gene and frequencies of the haplotypes in the global populations. (A) Relative map for the six SNPs examined. The SNPs examined in this study are numbered serially, and their rs numbers, alleles reported, and allele frequencies of JPT in the HapMap database are shown. For the SNP6, the amino acid change (Gly/Glu) has been reported in dbSNP. (B) Ancestral allele frequencies of SNPs in *PER2*. The allele frequency data from Japan are divided into four groups: subjects with physiological data, Northern Kyushu, Ryukyu, and JPT. Those are compared to three approximate geographic populations: East Asia (CHB, CHS), Europe (CEU, FIN, GBR, IBS, TSI) and Africa (LWK, YRI) from the 1000 Genomes Project database. (C) Haplotype frequencies of *PER2* in each geographical region. The numbers of local populations included in the geographical region are shown in parentheses. The N represents the numbers of the individuals. The combined frequencies of the remaining haplotype (Residuals) are less than 1.0% in all the geographical regions, indicating that each haplotype in the residual is extremely uncommon among the samples examined.

We selected six and four single nucleotide polymorphism (SNP) sites covering *PER2* and *PER3*, respectively (Fig 1A and S1 Fig). In the choice of the SNPs, we searched the transcriptional regulatory region, which was thought to fulfill an important role in PER oscillation, especially focusing on the *cis*-element motifs such as E-boxes. This was because subtle phenotypic differences among human populations were likely related to genetic polymorphisms found in the transcriptional regulatory regions. To predict the location of *cis*-elements, we performed a motif search using the program, TFSEARCH [32]. In *PER2*, three SNPs, supposed to exist in the motif, were found (SNP2, SNP3, and SNP4). However, even if a variation in the motif shows an association with the physiological variations, we could not determine whether or not the former is causative of the latter. We should consider length of the linkage between the variations found in a population. Therefore, we conducted haplotype analyses to clarify the relationships between physiological variations and the gene regions. SNPs were selected at a nearly equal physical distance so as to uniformly cover the whole gene. As a criterion of the SNP selection, we used the SNPs found in the JPT that had a minor allele frequency at above approximately 30%. Furthermore, because in the East Asian population the length of the haplotype block (i.e., the region where linkage disequilibrium [LD] is strong) is estimated to be 13.2 kb on average [33,34], we took that into consideration and chose SNPs to cover the whole gene region. The search was conducted using the dbSNP [35] and the browser of HapMap database [36].

SNP typing was performed using the TaqMan Genotyping Master Mix and TaqMan SNP genotyping assays according to the manufacturer’s recommended protocol (Applied Biosystems, Tokyo, Japan) on the LightCycler 480 System II (Roche Diagnostics, Tokyo, Japan). However, in the case of these SNPs in the *cis*-element motif, which did not satisfy the criterion of allele frequency, we conducted PCR-direct sequencing for the transcriptional regulatory region. PCR amplification was conducted using two pairs of primers: primer set 1 was *hPER2*_F1 (5'-TCC AGT GCC CCG CTT GAG TG-3') and *hPER2*_R1 (5'-CTT TGG GGT AGG TGG GTG GAG TG-3'), and primer set 2 was *hPER2*_F2 (5'-AGG CCG TCA GCA GCT CTA TGT C-3') and *hPER2*_R2 (5'-CAA CCA CAC GCA GCC AAA AC-3'). These primers were designed on the basis of the human reference genome sequence (GRCh37) extracted from the Ensemble database [37]. Approximately 50 ng of the genomic DNA was used as a template for PCR in a 25 *μ*l solution containing dNTP at 0.2 mM, 1.0 *μ*M of each of primer, 0.5 U of EX Taq HS polymerase (TaKaRa Shuzo Co., Kyoto, Japan), and the reaction buffer attached to the polymerase. Reactions were carried out in the PCR Thermal Cycler Dice (TaKaRa Shuzo) using the following protocol: in the primer set 1, an initial denaturing step at 94°C for 5 minutes, 35 cycles of denaturation at 94°C for 30 seconds, annealing at 66°C for 30 seconds, extension at 72°C for 10 seconds, and a final extension step at 72°C for 2 minutes; in the primer set 2, an initial denaturing step at 94°C for 5 minutes, 35 cycles of denaturation at 94°C for 30 seconds, annealing at 62°C for 30 seconds, extension at 72°C for 5 seconds, and a final extension step at 72°C for 2 minutes. The PCR products were diluted 20-fold and used as templates in the direct sequencing reaction. DNA sequencing was performed by the Sanger method using the BigDye Terminator v3.1 cycle sequencing kit according to the commercial protocol (Applied Biosystems), and was then analyzed in an ABI3130 or ABI3500 DNA Sequencer (Applied Biosystems). Base-calling and detection of heterozygotes were performed using the Sequencing Analysis Software v5.4 (Applied Biosystems) followed by visual inspection for SNPs. All variants were confirmed by reading both strands. Sequences were aligned with the SeqMan Pro software (DNASTAR, Madison, WI).

### Statistical analysis

For each SNP detected in any of the populations, we examined whether the Hardy-Weinberg equilibrium was kept at *P* > 0.05 by the χ^2^ test before performing any subsequent analyses. Haplotype estimation for individuals of each population was conducted using the PHASE algorithm [38] of DnaSP v5.10 [39]. The relationship between the physiological data and the allele, haplotype, and genotype was statistically analyzed. Statistical significance testing was performed by the Kruskal-Wallis test and Scheffe’s method of multiple comparison tests using the program R with Aoki’s R script (http://aoki2.si.gunma-u.ac.jp/R/src/kruskal_wallis.R, encoding = "euc-jp"). *P*-values < 0.05 were considered as statistically significant.

### Phylogenetic analysis and estimation of divergence time

To investigate the evolutionary relationship between the haplotypes, a phylogenetic network was reconstructed using the median-joining algorithm [40] of the NETWORK4.6.1.1 program (http://www.fluxus-engineering.com). Based on the genome sequences of chimpanzees (*Pan troglodyte*), gorillas (*Gorilla gorilla gorilla*), and macaques (*Macaca mulatta*) extracted from the Ensemble database, we estimated the ancestral alleles of each SNP.

The divergence times of *PER2* haplotypes were estimated. First, from the 1000 Genome Project (phase 3) database [41], we extracted the phased-variant data of the whole *PER2* region including each 2 kb of the 5' and 3' flanking regions for each individual that has three major haplotype (Hap1, Hap2, and Hap3) homozygotes in the geographical populations YRI, CEU, and JPT. Among 2,504 individuals, eight were chosen by the smallest ID number of each group (NA11894, NA18939, NA18486, NA07000, NA18942, NA18504, NA12282, and NA18945). At the same time, we got the corresponding sequence data from the human reference sequence GRCh37 that was used for mapping on the 1000 Genome Project (phase 3) database. The 16 chromosome sequences were acquired by rewriting the reference sequence for only the extracted SNP sites using the Python program. In addition, we downloaded the pairwise alignment data of the corresponding reference sequences for human (GRCh37) and chimpanzee (panTro4) [42–44] from UCSC Genome Browser database [45]. By using the data, we aligned the above 16 human sequences and the one homologous sequence of chimpanzee.

Next, a phylogenetic tree reconstruction and an estimation of evolutionary distance were performed using the MEGA v6.06 software [46]. A neighbor-joining (NJ) tree [47] for the intron region was constructed using the p-distance method [48]. All positions containing gaps and missing data were eliminated. Bootstrap analysis was performed using 1000 replications [49]. To assess the neutral mutation rate μ per nucleotide site per year for the *PER2* locus, the mean number of base substitutions per site over intron sequence pairs between human and chimpanzee (*d*_I_) was calculated using the Jukes-Cantor model [50], and the mean number of synonymous substitutions per synonymous site between human and chimpanzee (*d*_S_) was calculated using the Nei-Gojobori model [51]. Using the mean number of intron sites (*L*_I_) and that of synonymous sites (*L*_S_), the mean divergence between human and chimpanzee (*d*) was given: *d* = (*d*_I_*L*_I_ + *d*_S_*L*_S_)/(*L*_I_ + *L*_S_). By using the speciation time of humans and chimpanzees *T*_S_, we got *d* = 2*T*_S_μ. From the above two equations, we estimated μ of the *PER2* locus. Based on the mutation rate, we estimated the average divergence time of the sequence pairs in all the haplotypes (Hap1, Hap2, and Hap3) (*T*_*PER2*_), and especially in Hap1 (*T*_Hap1_). We calculated the average number of differences of all the sequence pairs between two clades bifurcated by the node of the most recent common ancestor for all the haplotypes (π_d-all_) and that for Hap1 samples (π_d-Hap1_). We used the average number of pairwise differences π_d_ with the Jukes and Cantor correction [50] between *PER2* intron sequences of humans. We subsequently estimated the times of *PER2* haplotypes from the equation *T* = π_d_/2μ by assuming a molecular clock.

## Results

### Physiological variations

We detected variations in the MEQ score as an index of chronotypes and the physiological variations in the percentage of pupil constriction and that of melatonin suppression as indices of circadian and non-image-forming effects of light, for our 43 subjects (Table 1). An individual with high melatonin suppression is thought to have a high light sensitivity [11].

**Table 1:** Summary of subjects’ physiological characteristics.

Characteristics	Number of individuals	Mean	SD
Age, y	43	21.02	1.37
MEQ score	39	46.97	6.77
Pupil constriction, %	41	0.71	0.07
Melatonin suppression, %	35	32.01	43.22

The MEQ scores of four subjects were excluded due to unanswered questions and/or incorrect answers. The melatonin data of eight subjects were excluded because melatonin concentration of seven subjects did not start to increase (>5.0 pg/ml) by the time of light exposure, and melatonin concentration of one subject started to decrease consecutively from 2 hours before light exposure. The pupil data of two subjects could not be measured correctly, therefore, they were excluded.

Although there was a large interindividual difference in raw data of melatonin concentration, the melatonin concentrations increased under dim light and they decreased after light exposure (S2 Fig). The melatonin concentration from samples taken at home at the same time, i.e., 3 hours after light exposure, was significantly higher than that before light exposure (0 h) (t = 3.98, *P* < 0.001) and 3 hours after light exposure (3 h) (t = 6.42, *P* < 0.001) on the experimental day. The results of percentage of melatonin concentration clearly showed that the increase in melatonin concentration started before light exposure. However, there was a large interindividual variation in percentages of melatonin suppression during light exposure. Therefore, we used the percentage of melatonin suppression at 3 hours after light exposure as an index of circadian photosensitivity in the following association studies.

### Allele/haplotype frequencies in *PER* genes

We chose six SNPs covering the *PER2* locus (44.6 kb): three were located in the putative promoter region of intron 1, and the others were in the 5' flanking region, intron 8, and exon 23 (Fig 1A). We also chose four SNPs located in introns 3, 7, 12, and in the 3' flanking region covering the *PER3* locus (60.5 kb), and examined them as well as in *PER2* (S1 Fig). In addition to the 43 subjects with physiological data, we genotyped the SNPs in *PER2* and *PER3* for 91 non-subjects (without physiological measurements). The SNP2, SNP3, and SNP4 in the putative promoter region of *PER2* were fixed in all the samples examined in this study, and we found no difference of the allele frequencies at the other SNPs between the subjects and non-subjects in both the *PER2* and *PER3* genes (Fig 1B and S3 Fig).

The haplotype frequencies were estimated based on phased haplotype data (Fig 1C and S4 Fig). We found that three haplotypes in *PER2*, Hap1 (TGGGCT), Hap2 (CGGGCC), and Hap3 (CGGGTC) accounted for more than 92% of the chromosomes in three populations (subjects, and non-subjects of Northern Kyushu and Ryukyu), and that three haplotypes in *PER3*, Hap1 (CCTT), Hap2 (AAGG), and Hap3 (ACTT) accounted for more than 96%. There was no difference between the subjects and non-subjects in haplotype frequency distribution, indicating that the subjects were not genetically deviated so that we could analyze them statistically without any sampling bias.

### Association between genetic polymorphisms and physiological variations

**Fig 2:**
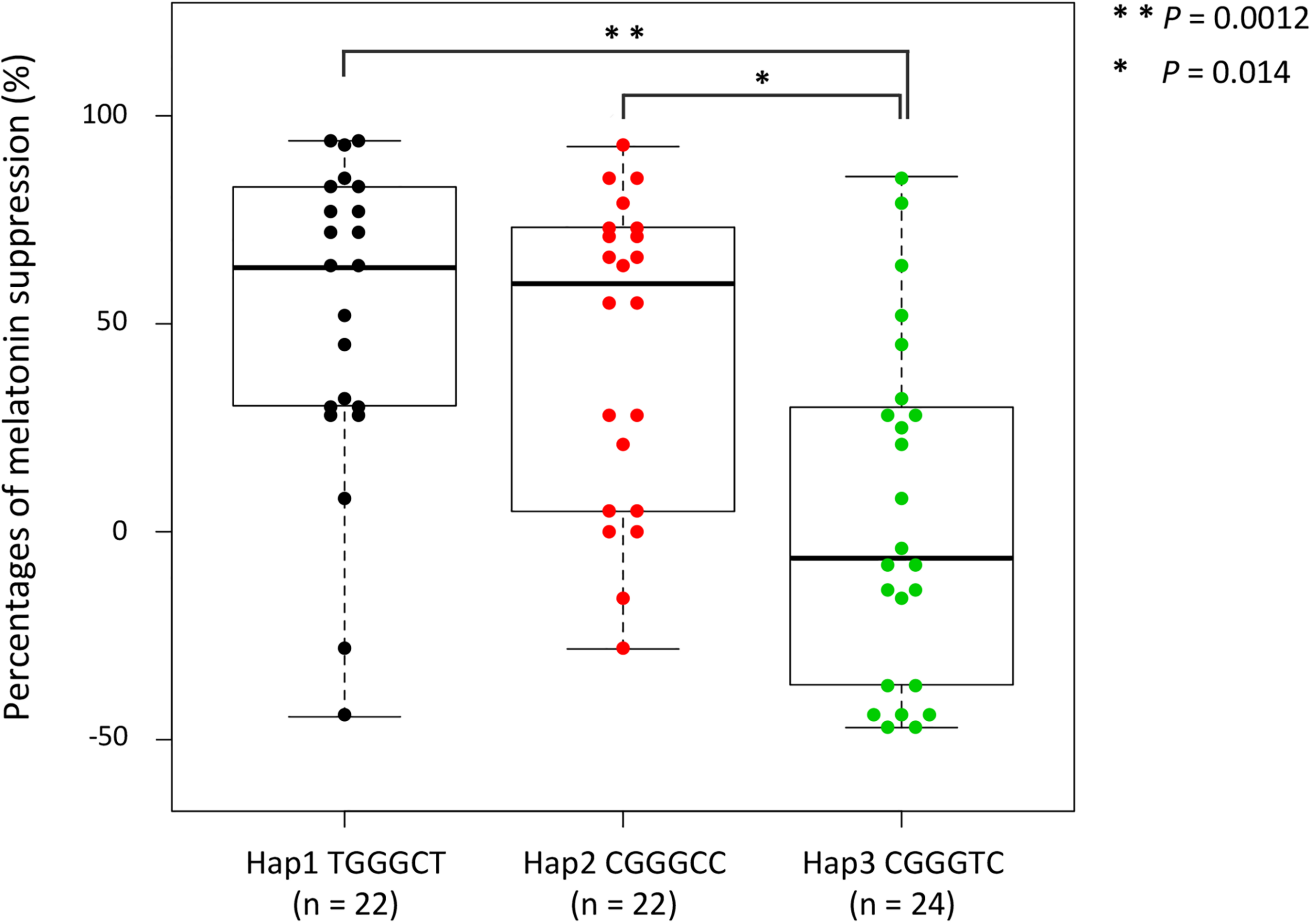
Comparison of the distributions of percentages of melatonin suppression for three major haplotypes of the *PER2* locus. Thick middle lines in the boxes represent the medians, the tops and bottoms of the boxes represent the third and the first quartiles, respectively, and the lower and upper error bars indicate the minimum and the maximum values. One dot represents one chromosome, and the numbers of chromosomes, n, are shown in parentheses. The Kruskal-Wallis test and Scheffe’s method of multiple comparison tests were performed, and the pairs of Hap1-Hap3 and Hap2-Hap3 revealed significant differences.

We examined associations between haplotypes of the *PER* genes and physiological variations in the response to light stimulus (Fig 2 and S5–S7 Figs). In *PER2*, the percentages of melatonin suppression of Hap1 (TGGGCT) and Hap2 (CGGGCC) were significantly different from that of Hap3 (CGGGTC) (*P* < 0.005 and *P* < 0.05, respectively) (Fig 2). Meanwhile, no association was found between the *PER3* haplotypes and the percentages of melatonin suppression (S5 Fig). Two other physiological variations (percentage of pupil constriction and MEQ score) were also examined to see if these variations were associated, but no association was found in either *PER2* or *PER3* (S6 and S7 Figs). Therefore, only the association between the *PER2* haplotypes and the percentages of melatonin suppression was shown in our series of examinations.

**Fig 3:**
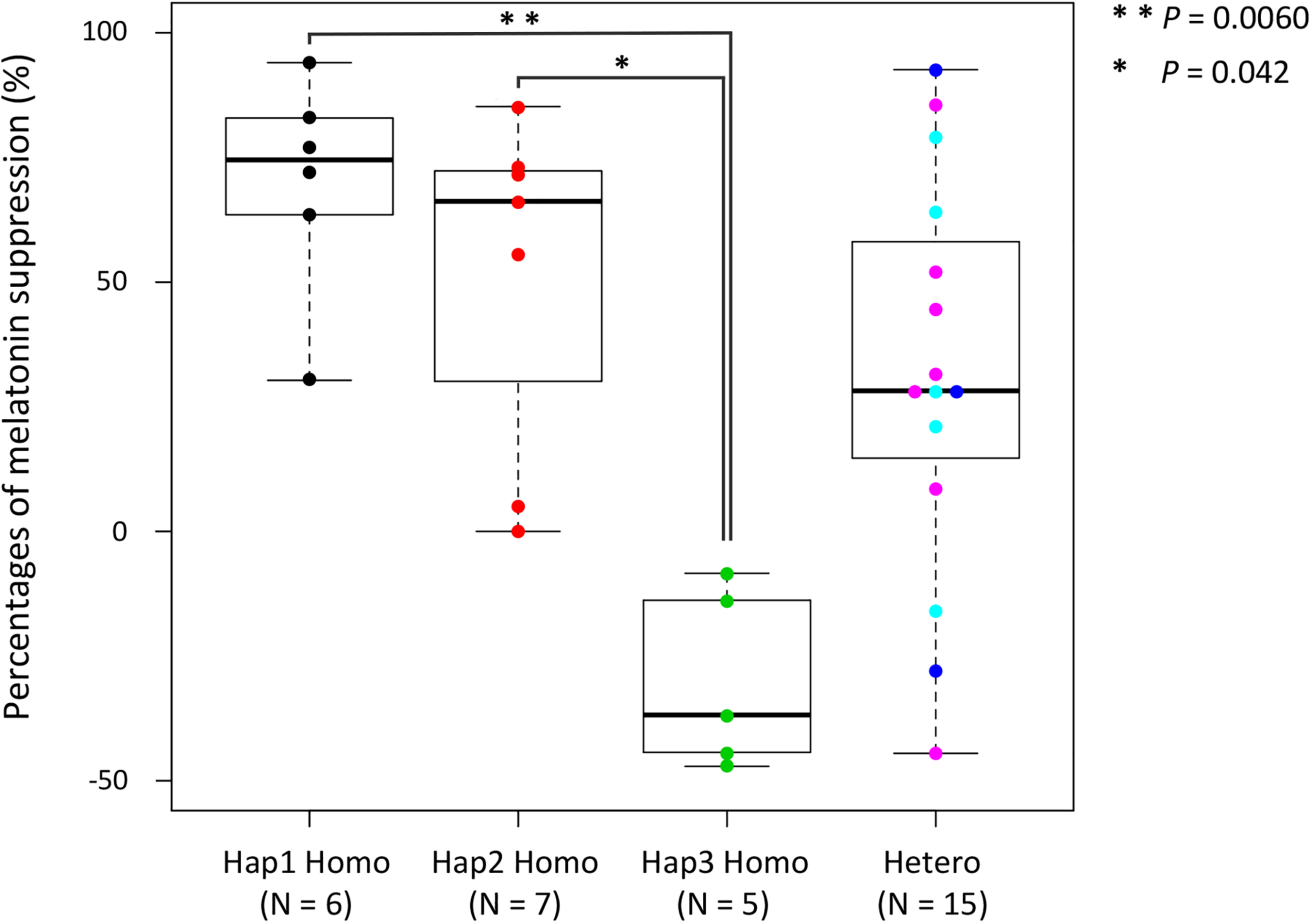
Comparison of the distributions of melatonin suppression for the genotypes of *PER2*. The thick middle lines in the boxes represent the medians, the tops and bottoms of the boxes represent the third and the first quartiles, respectively, and the lower and upper error bars indicate the minimum and the maximum values. One dot represents one subject, and the numbers of individuals, N, are shown in the parentheses. In the case of heterozygote, differently colored dots represent different genotypes. The Kruskal-Wallis test and Scheffe’s method of multiple comparison tests were conducted, and the pairs of Hap1-Hap3 and Hap2-Hap3 homozygotes showed significant differences.

In addition, we examined the association between the genotype and the phenotype. We also found that the percentages of melatonin suppression of Hap3 homozygotes were significantly different from those of Hap1 and Hap2 homozygotes in *PER2* (*P* < 0.01 and *P* < 0.05, respectively), whereas the heterozygotes varied in the ratios (Fig 3 and S8 Fig). Interestingly, the median of the percentage of melatonin suppression in the heterozygotes came to the intermediate point (28.2%) between the median of that of the Hap1 and Hap2 (71.4%) and that of the Hap3 (–36.8%), which likely indicates the Mendelian inheritance with incomplete dominance. All the SNPs in *PER2* showed strong associations with the percentage of melatonin suppression (S9 Fig), so that we could not identify which SNP was functionally causative of the melatonin suppression. Rather, this was likely to be attributed to a strong linkage between the SNPs examined.

### *PER2* haplotype frequency distribution and evolution

We then examined the allele frequencies of the six SNPs in *PER2* of 10 worldwide populations from the international database. We found the frequency differences between Africans and non-Africans in *PER2* (Fig 1B and S3 Fig). Three major haplotypes, Hap1, Hap2, and Hap3 accounted for more than 82% of the chromosomes in any worldwide populations, and Hap1 was absent from Africans, whereas Hap3 occupied more than 72%, suggesting a possibility that Hap1 appeared outside of Africa (Fig 1C and S4 Fig).

**Fig 4:**
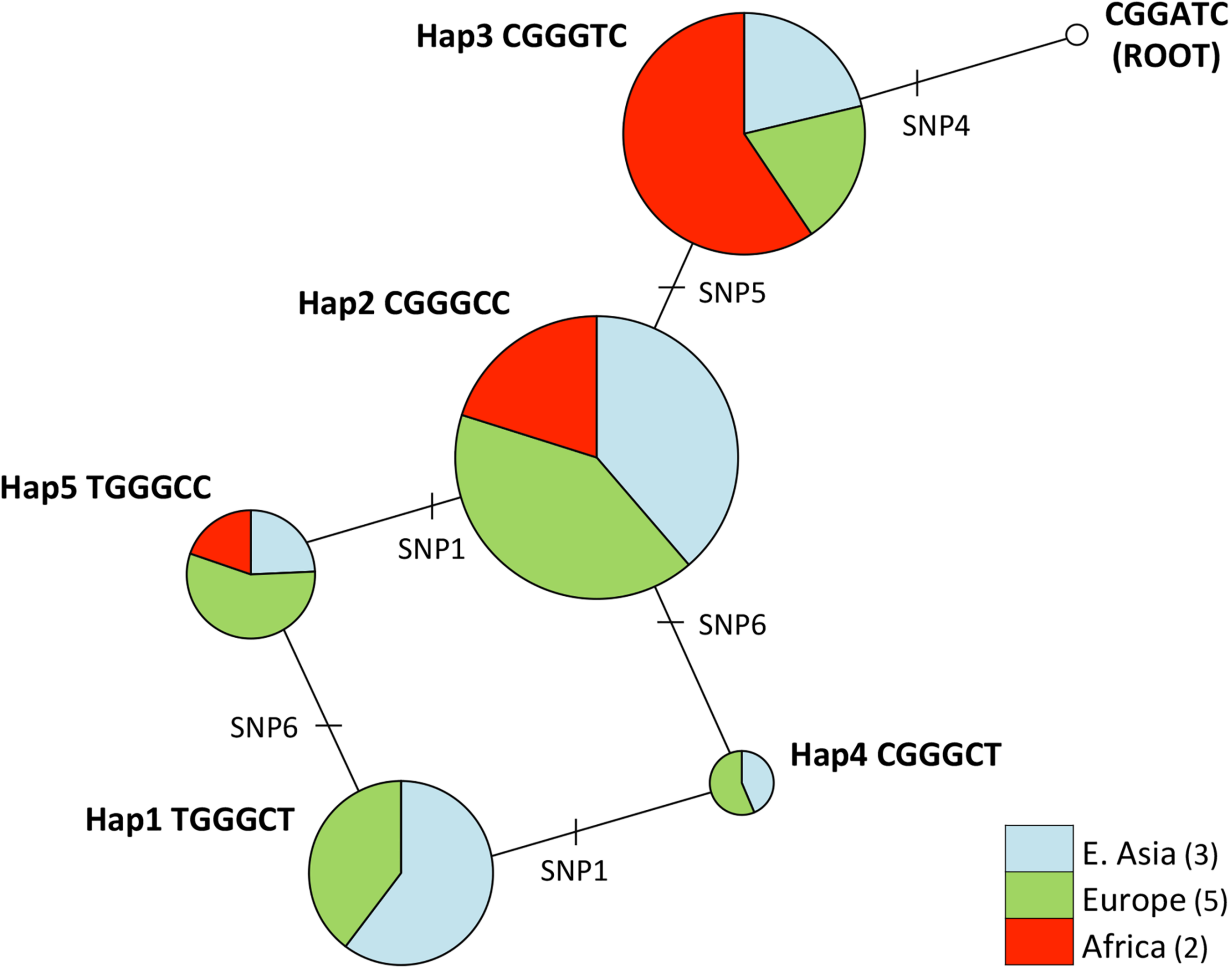
Phylogenetic network for *PER2* haplotypes. The circles represent the haplotypes, and the circle size is proportional to the sum of the number of chromosomes in all the populations for each haplotype. The pie charts show the haplotype frequencies in each geographical region: three populations from East Asia (JPT, CHB, CHS), five from Europe (CEU, FIN, GBR, IBS, TSI), and two from Africa (LWK, YRI). The minor haplotypes (<1.0%) are omitted here.

To see the evolutionary history of the *PER2* haplotypes, we constructed a phylogenetic network (Fig 4). The pie charts represent haplotype frequencies, and the size of the pie is proportional to the total number of chromosomes in the worldwide populations. The root was estimated to be CGGATC based on the nucleotide sequences from chimpanzee, gorilla, and macaque, which has a G to A substitution from Hap3 (CGGGTC) at SNP4, so that Hap3 was estimated to be an ancestral haplotype in humans. The Hap2 (CGGGCC) has a T to C substitution from Hap3, and was the most common (33.9%) in the worldwide populations. There was a reticulation from Hap2 to Hap1 (TGGGCT) via Hap4 (CGGGCT) or Hap5 (TGGGCC), indicating a track of possible recombination: Hap4, the uncommon haplotype in the world, would have appeared through the recombination event between Hap1 and Hap2. Thus, the phylogenetic network suggests that the low light-sensitive haplotype, Hap3, more frequently occupied by Africans was the ancestral haplotype, and the high light-sensitive haplotypes, Hap1 and Hap2, were the derived haplotypes from Hap3.

We estimated the divergence times of *PER2* haplotypes using nucleotide sequences of the 1000 Genome data [41]. We extracted the sequence data from YRI, CEU, and JPT who had homozygotes of three major haplotypes (Hap1, Hap2, and Hap3), and used chimpanzee sequence as the outgroup. For human and chimpanzee, the mean divergence over sequence pairs of intron (*d*_I_) and that of the synonymous site (*d*_S_) were *d*_I_ = 0.0159 and *d*_S_ = 0.0128, respectively, and the mean number of intron sites (*L*_I_) and that of the synonymous sites (*L*_S_) were *L*_I_ = 44,988 and *L*_S_ = 1,187, respectively. Based on these data, the weighted mean divergence between human and chimpanzee, *d* = 0.0158, was estimated. Considering the speciation time of humans and chimpanzees *T*_S_ as 6,000,000 years ago [52,53], we estimated the neutral mutation rate μ for the *PER2* locus was μ = 1.32 × 10^−9^ per nucleotide site per year. As a result of a phylogenetic tree based on the intron sequences, Hap1 was monophyletic (S10 Fig). To know the emergence time of all haplotypes and Hap1, we calculated each average nucleotide divergence: π_d-all_ = 0.109 × 10^−2^ and π_d-Hap1_ = 0.115 × 10^−3^. They are an average branch length at the deepest time in the tree and one at the deepest in the Hap1. From these estimates, we estimated the average divergence time of the sequence pairs in all the haplotypes at *T*_*PER2*_ = 415,000 years ago (KYA), and that in Hap1 at *T*_Hap1_ = 43.7 KYA.

However, in this calculation, we did not consider the possibility of recombination. To consider this possibility, we examined the incompatible pairs of segregating sites [54]. About 5% of all pairs of segregating sites showed incompatibility. Incompatible pairs of sites concentrated in a region ranging from 21,113 to 21,557. Those sites showed incompatibility not only within that region but also with sites in other adjacent regions (5' and 3' to the region). These incompatible pairs of sites were results of either recurrent mutations or recombinations. To avoid the effect of inclusion of these incompatible sites on the estimation of the divergence time, we excluded the regions showing incompatibility and divided the *PER2* region into upstream and downstream regions as independent loci. As a result, for the upstream, the average numbers of pairwise differences were calculated: π_d-all_ = 0.566 × 10^−3^ and π_d-Hap1_ = 0.111 × 10^−3^. The average divergence times of the sequence pairs were estimated to be: *T*_*PER2*_ = 215 KYA, and *T*_Hap1_ = 42.0 KYA. For the downstream, the average numbers of pairwise differences were calculated: π_d-all_ = 0.134 × 10^−2^, and π_d-Hap1_ = 0.135 × 10^−4^. The average divergence times of the sequence pairs were estimated to be: *T*_*PER2*_ = 510 KYA, and *T*_Hap1_ = 5.11 KYA. But the upstream and downstream regions of *PER2* were compatible to each other. Especially there was only one informative site in the upstream region and no informative sites in the downstream region of Hap1. There was no evidence that the two loci had independent evolutionary histories. Thus, we combined the two regions and calculated the average numbers of pairwise differences: π_d-all_ = 0.985 × 10^−3^, and π_d-Hap1_ = 0.582 × 10^−4^. The average divergence times of the sequence pairs were estimated to be: *T*_*PER2*_ = 374 KYA, and *T*_Hap1_ = 22.1 KYA (S10 Fig). These results suggested that, even though we considered the recombinations, Hap2 and Hap3 had already existed before the Out-of-Africa migration that occurred approximately 50 KYA [55–58], while after the migration, Hap1 appeared and the *PER2* haplotype variations increased.

## Discussion

Our genetic analysis on the subjects with physiological data revealed that the individual variations in the physiological reaction for light-stimulus are significantly associated with the *PER2* polymorphisms. This is an unexpected result. Melatonin suppression is observed in an artificial circumstance of light exposure during nighttime. Therefore, it has been used as a measure of plastically circadian sensitivity to light stimulus [9]. On the other hand, the internal circadian rhythm is generated by the autoregulatory transcriptional negative-feedback loop of clock gene expression, and maintained even in constant darkness artificially provided in an experimental laboratory [59]. The circadian rhythm, which is regulated in a suprachiasmatic nucleus (SCN), synchronizes to an external 24-hour day/night cycle through intrinsically photosensitive retinal ganglion cells of the retina [60], and the information is sent to the pineal gland, which secretes melatonin [3,61]. Related to clock genes, the suppression of melatonin secretion, however, has not drawn much attention. In the present study, we show that the variation of the percentages of melatonin suppression is significantly associated with the *PER2* haplotype/genotype. In this association analysis, we used the percentage of melatonin suppression at 3 hours after light exposure as an index of circadian photosensitivity. In this case, if the light exposure was timed late after the offset of melatonin synthesis regardless of adjusting the start time of the exposure for each subject, melatonin secretion could decrease either due to light-induced suppression itself or due to the natural reduction in salivary concentration at 3 hours after light exposure. Therefore, we confirmed that light exposure was properly timed in all the subjects (S2 and S8 Figs). In addition, the average of percentages of melatonin suppression by light exposure for 3 hours was also used as the index for these analyses. We found that there was nearly the same tendency as that in Fig 3, and the percentages of melatonin suppression of Hap3 homozygotes were significantly different from those of Hap1 homozygotes in *PER2* (*P* < 0.01), while those of Hap3 homozygotes did not show a significant difference from those of Hap2 homozygotes. However, that result is likely due to the effect of the small sample size for the association analysis, therefore, re-examination using a larger sample population is warranted in future studies. In the present study, we also show that *PER2* haplotypes are not associated with pupillary reflex. These results suggest that there are different systems driving light-induced pupil constriction and light-induced melatonin suppression, which has been discussed in the literature [62, 63]. SCN mediates light-induced melatonin suppression but not light-induced pupillary constriction, which is mediated by the olivary pretectal nucleus [60, 64]. Therefore, in the present study there was no significant correlation between percentages of light-induced melatonin suppression and pupil constriction. These findings suggest that the functions and/or the expression levels of the PER2 protein are more significantly associated with low-sensitivity to light in Hap3 than in the other haplotypes.

A linkage analysis shows that one of the variants of *PER2* (S662G) is associated with the sleep-phase disorder: FASPS (familial advanced sleep-phase syndrome) [17], and an empirical study using mice harboring the PER2 S662G mutation shows that their circadian rhythmicity has been changed [65]. Many other reports also show the associations between *PER2* polymorphisms and diurnal preference [66–71]. A recent genome-wide association study (GWAS), using 89,283 individuals, reports that the *PER2* is one of the loci associated with self-reported morningness [72]. These studies suggest that the *PER2* polymorphisms produce phenotypic variations of circadian rhythmicity. I.e., a sleep-phase disorder or an extreme phenotype is caused by a particular haplotype of *PER2* that has a strong effect on circadian rhythmicity, while for the other haplotypes with weak effects, the phenotype differences have been recognized as physiological variations. Our findings support this prediction. I.e., the *PER2* polymorphisms may affect circadian phase shifting variations related to the percentage of melatonin suppression [10]. A previous study of *PER3* reported a relationship between one VNTR polymorphism in exon 18 of the *PER3* gene and melatonin suppression [73]. On the other hand, we have not found any relationships between the *PER3* haplotypes and the percentages of melatonin suppression in the present study (S5B Fig). Supporting our results, some previous studies using mice or cultured cells have shown that *mPer2* is important for light induced resetting of the circadian clock [74,75], and *mPer2* mRNA expression in SCN is induced by brief light exposure in constant darkness [76], but *mPer3* has lost responsiveness to light and is not necessary for maintaining the circadian rhythmicity [77]. In our candidate-gene approach, therefore, the significant association between the *PER2* polymorphism and the percentage of melatonin suppression in humans has been clearly shown.

It is unclear how the PER2 protein is related to the mechanisms of melatonin suppression. Three SNPs in the transcriptional regulatory region have been fixed in almost all human populations (Fig 1B), suggesting that the mutations which occurred in this region have been critically concerned with survival throughout its evolutionary history. The SNP6, non-synonymous substitution in exon 23, does not separate high and low light-sensitive haplotypes, but the SNP5 in intron 8 does (Figs 1A and 4). The haplotypes that have a C allele at SNP5 show higher percentages of melatonin suppression; while the haplotype that has T allele, Hap3, shows lower percentages (Figs 2 and 4). There are two possibilities for this phenomenon: one is that SNP5 itself directly contributes to the phenotype. In this case, there should be unknown transcriptional-regulation factors around SNP5 in intron 8. Another possibility is an effect of linkage between SNP5 and the other SNPs not examined in the present study. Some previous studies show there exists a PAS (PER-ARNT-SIM) domain (exon 9 to exon 11) near the SNP5, CK1ε binding region (exon 15 to exon 18), etc., indicating this region is very important for protein-protein interaction of the product of the *PER2* gene, and is essential for maintaining circadian rhythmicity [78,79]. To elucidate this, a more detailed SNP search by resequencing the subjects and functional analysis of each haplotype using cells would be required.

Our haplotype analyses have shown that the low light-sensitive haplotype is more frequently found in Africa than outside Africa, and that non-African populations have a larger number of haplotypes than do African populations (Fig 1C). Phylogenetic network analysis has shown that the low light-sensitive haplotype is the ancestral type and that the high light-sensitive haplotypes are the derived types; the divergence time of three major haplotypes, *T*_*PER2*_, is estimated to be 374 KYA. Considering the Out-of-Africa migration scenario in which anatomically modern humans appeared in Africa around 200 to 100 KYA and migrated out of Africa approximately 50 KYA, the estimate of all haplotype divergence time suggests that the haplotypes emerged almost concurrently with, or earlier than the origin of anatomically modern humans in Africa. The divergence time of Hap1 as high light-sensitive haplotype, which has been estimated to be 22.1 KYA, coincides with, or a little later than the period when humans migrated out of Africa. This suggests that the high light-sensitive haplotypes have increased in the world after the Out-of-Africa migration of modern humans, which is a totally opposite pattern commonly observed in most of the regions of the human genome that African populations have more haplotypes than do non-African populations [80–82]. The common pattern is explained by the Out-of-Africa migration. Because of the bottleneck effect caused by the Out-of-Africa event, African populations have more diversity than do non-Africans [83,84]. Therefore, when looking at a part of the human genome, the number of haplotypes in African populations is greater than that of non-African populations in most of the regions of the entire genome.

To adequately explain the uncommon pattern generated from our results requires three different scenarios. First, it can be explained by neutrality: a small number of haplotypes in Africa and more haplotypes outside of Africa occurred by chance. Second, a strong selective pressure prevented changes in this gene locus in Africa: therefore, Africans have high frequencies of Hap3 at more than 72%. But the pressure weakened and became neutral in regions outside of Africa, and the other haplotypes increased and spread throughout the world concurrent to and following the Out-of-Africa migration. And third, a balancing selection, in which more haplotypes were advantageous, worked outside of Africa. This could be a possible reason the three haplotypes maintain nearly the same frequencies among non-African populations, though it is very difficult to prove if this frequency pattern is due to the balancing selection. Modern humans have spread into vast geographical regions in which there are huge variations in light environments. We revealed the clock gene *PER2* polymorphisms account for the physiological variation of melatonin suppression as circadian light sensitivity, and further studies of a greater number of sequences, probably using next generation sequencing (NGS), are required to elucidate the evolutionary history of the clock gene network system in humans.

## Supporting information

S1 Fig Relative maps for the SNPs examined in this study. (A) Six SNPs on *PER2* and (B) four SNPs on *PER3* are numbered serially, and their rs numbers, alleles reported, and allele frequencies of JPT in the HapMap database are shown respectively.
(TIF)

S2 Fig Melatonin profiles of the subjects. (A) The raw data of melatonin concentration in all the subjects. The bold line shows the average. The start time of light exposure (0 h) was individually set 3 and 3.5 hours before the midpoint of sleep of each subject. There was a large interindividual difference. The dots shows the melatonin concentration measured at home at the same time as 3 hours after light exposure (3 h) 2 days before the experiment. (B) The percentage of melatonin concentration (left vertical axis) and the percentage of melatonin suppression by light (right vertical axis), which were calculated based on the data before light exposure (0 h). Although melatonin concentration increased during dim light in all the subjects, there was a large interindividual difference in the percentage of melatonin suppression.
(TIF)

S3 Fig **Ancestral allele frequencies of SNPs (A) in *PER2* and (B) in *PER3*.** Three groups, Subjects, Northern Kyushu and Ryukyu were genotyped in this study. N represents the number of individuals.
(TIF)

S4 Fig **Haplotype frequencies for (A) *PER2* and (B) *PER3*.** The numbers of local populations included in the geographical region are shown in parentheses. N represents the numbers of the individuals. The combined frequencies of the remaining haplotype (Residuals) are less than 1.0% in all the geographical regions.
(TIF)

S5 Fig **Comparison of the distributions of percentages of melatonin suppression for major haplotypes of (A) *PER2* and (B) *PER3*.** The thick middle lines in the boxes represent the medians, and the tops and bottoms of the boxes represent the third and the first quartiles, respectively. One dot represents one chromosome, and the numbers of chromosomes, n, are shown in parentheses.
(TIF)

S6 Fig **Comparison of the distributions of (A) percentages of pupil constriction and (B) MEQ score for three major haplotypes of *PER2*.** Thick middle lines in the boxes represent the medians, and the tops and bottoms of the boxes represent the third and the first quartiles, respectively. One dot represents one chromosome, and the numbers of chromosomes, n, are shown in parentheses.
(TIF)

S7 Fig **Comparison of the distributions of (A) percentages of pupil constriction and (B) MEQ score for four major haplotypes of *PER3*.** The thick middle lines in the boxes represent the medians, and the tops and bottoms of the boxes represent the third and the first quartiles, respectively. One dot represents one chromosome, and the numbers of chromosomes, n, are shown in parentheses.
(TIF)

S8 Fig Difference of changes in percentages of melatonin suppression between the genotypes of *PER2*. The percentages of melatonin suppression by light exposure in Hap1, Hap2, and Hap3 homozygotes. The data are shown as the mean + SD.
(TIF)

S9 Fig **Comparison of the distributions of percentages of melatonin suppression for *PER2* alleles of (A) SNP1, (B) SNP5, and (C) SNP6.** The thick middle lines in the boxes represent the medians, and the tops and bottoms of the boxes represent the third and the first quartiles, respectively. One dot represents one chromosome, and the numbers of chromosomes, n, are shown in parentheses. The Kruskal-Wallis test shows statistically significant differences.
(TIF)

S10 Fig An NJ tree for the *PER2* intron sequences and the divergence times of the haplotypes with 1000 bootstrap replicates. The phased sequences that had homozygotes of three major haplotypes (Hap1, Hap2, and Hap3) in YRI, CEU, and JPT were obtained from the 1000 Genome Project (phase 3) database. The arrows represent divergence times.
(TIF)

## References

[1] Czeisler CA, Allan JS, Strogatz SH, Ronda JM, Sanchez R, Rios CD, et al Bright light resets the human circadian pacemaker independent of the timing of the sleep-wake cycle. Science. 1986;233(4764): 667–671. doi: 10.1126/science.3726555 3726555 10.1126/science.3726555

[2] Reppert SM, Weaver DR. Coordination of circadian timing in mammals. Nature. 2002;418(6901): 935–941. doi: 10.1038/nature00965 12198538 10.1038/nature00965

[3] Khalsa SB, Jewett ME, Cajochen C, Czeisler CA. A phase response curve to single bright light pulses in human subjects. J Physiol. 2003;549(Pt 3): 945–952. doi: 10.1113/jphysiol.2003.040477 12717008 10.1113/jphysiol.2003.040477 PMC2342968

[4] Cajochen C. Alerting effects of light. Sleep Med Rev. 2007;11(6): 453–464. doi: 10.1016/j.smrv.2007.07.009 17936041 10.1016/j.smrv.2007.07.009

[5] Gamlin PDR, McDougal DH, Pokorny J, Smith VC, Yau KW, Dacey DM. Human and macaque pupil responses driven by melanopsin-containing retinal ganglion cells. Vision Res. 2007;47(7): 946–954. doi: 10.1016/j.visres.2006.12.015 17320141 10.1016/j.visres.2006.12.015 PMC1945238

[6] Lewy AJ, Wehr TA, Goodwin FK, Newsome DA, Markey SP. Light suppresses melatonin secretion in humans. Science. 1980;210(4475): 1267–1269. doi: 10.1126/science.7434030 7434030 10.1126/science.7434030

[7] Daneault V, Dumont M, Massé É, Vandewalle G, Carrier J. Light-sensitive brain pathways and aging. J Physiol Anthropol. 2016;35: 9 doi: 10.1186/s40101-016-0091-9 26980095 10.1186/s40101-016-0091-9 PMC4791759

[8] Lockley SW, Gooley JJ. Circadian photoreception: spotlight on the brain. Curr Biol. 2006;16(18): R795–797. doi: 10.1016/j.cub.2006.08.039 16979545 10.1016/j.cub.2006.08.039

[9] Arendt J. Melatonin and human rhythms. Chronobiol Int. 2006;23(1–2): 21–37. doi: 10.1080/07420520500464361 16687277 10.1080/07420520500464361

[10] Gooley JJ, Chamberlain K, Smith KA, Khalsa SB, Rajaratnam SM, Van Reen E, et al Exposure to room light before bedtime suppresses melatonin onset and shortens melatonin duration in humans. J Clin Endocrinol Metab. 2011;96(3): E463–472. doi: 10.1210/jc.2010-2098 21193540 10.1210/jc.2010-2098 PMC3047226

[11] Laakso ML, Porkka-Heiskanen T, Stenberg D, Alila A. Interindividual differences in the responses of serum and salivary melatonin to light In: Fraschini F, Reiter RJ, editors. Role of melatonin and pineal peptides in neuroimmunomodulation. New York: Plenum Press; 1991 pp. 307–311.

[12] Hébert M, Martin SK, Lee C, Eastman CI. The effects of prior light history on the suppression of melatonin by light in humans. J Pineal Res. 2002;33(4): 198–203. doi: 10.1034/j.1600-079X.2002.01885.x 12390501 10.1034/j.1600-079x.2002.01885.x PMC3925650

[13] Higuchi S, Motohashi Y, Ishibashi K, Maeda T. Less exposure to daily ambient light in winter increases sensitivity of melatonin to light suppression. Chronobiol Int. 2007;24(1): 31–41. doi: 10.1080/07420520601139805 17364578 10.1080/07420520601139805

[14] Higuchi S, Motohashi Y, Ishibashi K, Maeda T. Influence of eye colors of Caucasians and Asians on suppression of melatonin secretion by light. Am J Physiol Regul Integr Comp Physiol. 2007;292(6): R2352–2356. doi: 10.1152/ajpregu.00355.2006 17332164 10.1152/ajpregu.00355.2006

[15] Higuchi S, Nagafuchi Y, Lee SI, Harada T. Influence of light at night on melatonin suppression in children. J Clin Endocrinol Metab. 2014;99(9): 3298–3303. doi: 10.1210/jc.2014-1629 24840814 10.1210/jc.2014-1629

[16] Yasukouchi A. A perspective on the diversity of human adaptability. J Physiol Anthropol Appl Human Sci. 2005;24(3): 243–247. doi: 10.2114/jpa.24.243 15930815 10.2114/jpa.24.243

[17] Toh KL, Jones CR, He Y, Eide EJ, Hinz WA, Virshup DM, et al An h*Per2* phosphorylation site mutation in familial advanced sleep phase syndrome. Science. 2001;291(5506): 1040–1043. doi: 10.1126/science.1057499 11232563 10.1126/science.1057499

[18] Ebisawa T. Circadian rhythms in the CNS and peripheral clock disorders: human sleep disorders and clock genes. J Pharmacol Sci. 2007;103(2): 150–154. doi: 10.1254/jphs.FMJ06003X5 17299246 10.1254/jphs.fmj06003x5

[19] Takahashi JS, Hong HK, Ko CH, McDearmon EL. The genetics of mammalian circadian order and disorder: implications for physiology and disease. Nat Rev Genet. 2008;9(10): 764–775. doi: 10.1038/nrg2430 18802415 10.1038/nrg2430 PMC3758473

[20] Ukai H, Ueda HR. Systems biology of mammalian circadian clocks. Annu Rev Physiol. 2010;72: 579–603. doi: 10.1146/annurev-physiol-073109-130051 20148689 10.1146/annurev-physiol-073109-130051

[21] Hut RA, Paolucci S, Dor R, Kyriacou CP, Daan S. Latitudinal clines: an evolutionary view on biological rhythms. Proc Biol Sci. 2013;280(1765): 20130433 doi: 10.1098/rspb.2013.0433 23825204 10.1098/rspb.2013.0433 PMC3712436

[22] Cruciani F, Trombetta B, Labuda D, Modiano D, Torroni A, Costa R, et al Genetic diversity patterns at the human clock gene *period 2* are suggestive of population-specific positive selection. Eur J Hum Genet. 2008;16(12): 1526–1534. doi: 10.1038/ejhg.2008.105 18575464 10.1038/ejhg.2008.105

[23] Nadkarni NA, Weale ME, von Schantz M, Thomas MG. Evolution of a length polymorphism in the human *PER3* gene, a component of the circadian system. J Biol Rhythms. 2005;20(6): 490–499. doi: 10.1177/0748730405281332 16275768 10.1177/0748730405281332

[24] Ciarleglio CM, Ryckman KK, Servick SV, Hida A, Robbins S, Wells N, et al Genetic differences in human circadian clock genes among worldwide populations. J Biol Rhythms. 2008;23(4): 330–340. doi: 10.1177/0748730408320284 18663240 10.1177/0748730408320284 PMC2579796

[25] Costa R, Peixoto AA, Barbujani G, Kyriacou CP. A latitudinal cline in a *Drosophila* clock gene. Proc Biol Sci. 1992;250(1327): 43–49. doi: 10.1098/rspb.1992.0128 1361061 10.1098/rspb.1992.0128

[26] Rosato E, Peixoto AA, Costa R, Kyriacou CP. Linkage disequilibrium, mutational analysis and natural selection in the repetitive region of the clock gene, *period*, in *Drosophila melanogaster*. Genet Res. 1997;69(2): 89–99. 10191718 10.1017/s001667239700267x

[27] Sawyer LA, Sandrelli F, Pasetto C, Peixoto AA, Rosato E, Costa R, et al The *period* gene Thr-Gly polymorphism in Australian and African *Drosophila melanogaster* populations: implications for selection. Genetics. 2006;174(1): 465–480. doi: 10.1534/genetics.106.058792 16849607 10.1534/genetics.106.058792 PMC1569780

[28] 1000 Genomes Project Consortium. An integrated map of genetic variation from 1,092 human genomes. Nature. 2012;491(7422): 56–65. doi: 10.1038/nature11632 23128226 10.1038/nature11632 PMC3498066

[29] Horne JA, Ostberg O. A self-assessment questionnaire to determine morningness-eveningness in human circadian rhythms. Int J Chronobiol. 1976;4(2): 97–110. 102738 1027738

[30] Ishihara K, Saitoh T, Inoue Y, Miyata Y. Validity of the Japanese version of the Morningness-Eveningness Questionnaire. Percept Mot Skills. 1984;59(3): 863–866. doi: 10.2466/pms.1984.59.3.863

[31] Martin SK, Eastman CI. Sleep logs of young adults with self-selected sleep times predict the dim light melatonin onset. Chronobiol Int. 2002;19(4): 695–707. doi: 10.1081/CBI-120006080 12182497 10.1081/cbi-120006080

[32] Heinemeyer T, Wingender E, Reuter I, Hermjakob H, Kel AE, Kel OV, et al Databases on transcriptional regulation: TRANSFAC, TRRD and COMPEL. Nucleic Acids Res. 1998;26(1): 362–367. doi: 10.1093/nar/26.1.362 9399875 10.1093/nar/26.1.362 PMC147251

[33] Gabriel SB, Schaffner SF, Nguyen H, Moore JM, Roy J, Blumenstiel B, et al The structure of haplotype blocks in the human genome. Science. 2002;296(5576): 2225–2229. doi: 10.1126/science.1069424 12029063 10.1126/science.1069424

[34] International HapMap Consortium. A haplotype map of the human genome. Nature. 2005;437(7063): 1299–1320. doi: 10.1038/nature04226 16255080 10.1038/nature04226 PMC1880871

[35] Sherry ST, Ward MH, Kholodov M, Baker J, Phan L, Smigielski EM, et al dbSNP: the NCBI database of genetic variation. Nucleic Acids Res. 2001;29(1): 308–311. doi: 10.1093/nar/29.1.308 11125122 10.1093/nar/29.1.308 PMC29783

[36] International HapMap 3 Consortium. Integrating common and rare genetic variation in diverse human populations. Nature. 2010;467(7311): 52–58. doi: 10.1038/nature09298 20811451 10.1038/nature09298 PMC3173859

[37] Flicek P, Amode MR, Barrell D, Beal K, Billis K, Brent S, et al Ensembl 2014. Nucleic Acids Res. 2014;42(Database issue): D749–755. doi: 10.1093/nar/gkt1196 24316576 10.1093/nar/gkt1196 PMC3964975

[38] Stephens M, Smith NJ, Donnelly P. A new statistical method for haplotype reconstruction from population data. Am J Hum Genet. 2001;68(4): 978–989. doi: 10.1086/319501 11254454 10.1086/319501 PMC1275651

[39] Librado P, Rozas J. DnaSP v5: a software for comprehensive analysis of DNA polymorphism data. Bioinformatics. 2009;25(11): 1451–1452. doi: 10.1093/bioinformatics/btp187 19346325 10.1093/bioinformatics/btp187

[40] Bandelt HJ, Forster P, Röhl A. Median-joining networks for inferring intraspecific phylogenies. Mol Biol Evol. 1999;16(1): 37–48. doi: 10.1093/oxfordjournals.molbev.a026036 10331250 10.1093/oxfordjournals.molbev.a026036

[41] 1000 Genomes Project Consortium. A global reference for human genetic variation. Nature. 2015;526(7571): 68–74. doi: 10.1038/nature15393 26432245 10.1038/nature15393 PMC4750478

[42] Chiaromonte F, Yap V, Miller W. Scoring pairwise genomic sequence alignments. Pac Symp Biocomput. 2002;7: 115–126. 11928468 10.1142/9789812799623_0012

[43] Kent WJ, Baertsch R, Hinrichs A, Miller W, Haussler D. Evolution's cauldron: duplication, deletion, and rearrangement in the mouse and human genomes. Proc Natl Acad Sci U S A. 2003;100(20): 11484–11489. doi: 10.1073/pnas.1932072100 14500911 10.1073/pnas.1932072100 PMC208784

[44] Schwartz S, Kent WJ, Smit A, Zhang Z, Baertsch R, Hardison RC, et al Human–mouse alignments with BLASTZ. Genome Res. 2003;13(1): 103–107. doi: 10.1101/gr.809403 12529312 10.1101/gr.809403 PMC430961

[45] Kent WJ, Sugnet CW, Furey TS, Roskin KM, Pringle TH, Zahler AM, et al The human genome browser at UCSC. Genome Res. 2002;12(6): 996–1006. doi: 10.1101/gr.229102 12045153 10.1101/gr.229102 PMC186604

[46] Tamura K, Stecher G, Peterson D, Filipski A, Kumar S. MEGA6: molecular evolutionary genetics analysis version 6.0. Mol Biol Evol. 2013;30(12): 2725–2729. doi: 10.1093/molbev/mst197 24132122 10.1093/molbev/mst197 PMC3840312

[47] Saitou N, Nei M. The neighbor-joining method: a new method for reconstructing phylogenetic trees. Mol Biol Evol. 1987;4(4): 406–425. 3447015 10.1093/oxfordjournals.molbev.a040454

[48] Nei M, Kumar S. Molecular evolution and phylogenetics. New York: Oxford University Press; 2000.

[49] Felsenstein J. Confidence limits on phylogenies: an approach using the bootstrap. Evolution. 1985;39(4): 783–791. doi: 10.1111/j.1558-5646.1985.tb00420.x 28561359 10.1111/j.1558-5646.1985.tb00420.x

[50] Jukes TH, Cantor CR. Evolution of protein molecules In: Munro HN, editor. Mammalian protein metabolism. New York: Academic Press; 1969 pp. 21–132.

[51] Nei M, Gojobori T. Simple methods for estimating the numbers of synonymous and nonsynonymous nucleotide substitutions. Mol Biol Evol. 1986;3(5): 418–426. 3444411 10.1093/oxfordjournals.molbev.a040410

[52] Satta Y, Hickerson M, Watanabe H, O'hUigin C, Klein J. Ancestral population sizes and species divergence times in the primate lineage on the basis of intron and BAC end sequences. J Mol Evol. 2004;59(4): 478–487. doi: 10.1007/s00239-004-2639-2 15638459 10.1007/s00239-004-2639-2

[53] Scally A, Dutheil JY, Hillier LW, Jordan GE, Goodhead I, Herrero J, et al Insights into hominid evolution from the gorilla genome sequence. Nature. 2012;483(7388): 169–175. doi: 10.1038/nature10842 22398555 10.1038/nature10842 PMC3303130

[54] Takahata N, Satta Y. Selection, convergence, and intragenic recombination in HLA diversity. Genetica. 1998;102/103: 157–169. doi: 10.1023/A:1017029613342 9720277

[55] Gutenkunst RN, Hernandez RD, Williamson SH, Bustamante CD. Inferring the joint demographic history of multiple populations from multidimensional SNP frequency data. PLoS Genet. 2009;5(10): e1000695 doi: 10.1371/journal.pgen.1000695 19851460 10.1371/journal.pgen.1000695 PMC2760211

[56] Gravel S, Henn BM, Gutenkunst RN, Indap AR, Marth GT, Clark AG, et al Demographic history and rare allele sharing among human populations. Proc Natl Acad Sci U S A. 2011;108(29): 11983–11988. doi: 10.1073/pnas.1019276108 21730125 10.1073/pnas.1019276108 PMC3142009

[57] Gronau I, Hubisz MJ, Gulko B, Danko CG, Siepel A. Bayesian inference of ancient human demography from individual genome sequences. Nat Genet. 2011;43(10): 1031–1034. doi: 10.1038/ng.937 21926973 10.1038/ng.937 PMC3245873

[58] Harris K, Nielsen R. Inferring demographic history from a spectrum of shared haplotype lengths. PLoS Genet. 2013;9(6): e1003521 doi: 10.1371/journal.pgen.1003521 23754952 10.1371/journal.pgen.1003521 PMC3675002

[59] Wright KP Jr, Hughes RJ, Kronauer RE, Dijk DJ, Czeisler CA. Intrinsic near-24-h pacemaker period determines limits of circadian entrainment to a weak synchronizer in humans. Proc Natl Acad Sci U S A. 2001;98(24): 14027–14032. doi: 10.1073/pnas.201530198 11717461 10.1073/pnas.201530198 PMC61161

[60] Berson DM, Dunn FA, Takao M. Phototransduction by retinal ganglion cells that set the circadian clock. Science. 2002;295(5557): 1070–1073. doi: 10.1126/science.1067262 11834835 10.1126/science.1067262

[61] Møller M, Baeres FM. The anatomy and innervation of the mammalian pineal gland. Cell Tissue Res. 2002;309(1): 139–150. doi: 10.1007/s00441-002-0580-5 12111544 10.1007/s00441-002-0580-5

[62] Higuchi S, Ishibashi K, Aritake S, Enomoto M, Hida A, Tamura M, et al Inter-individual difference in pupil size correlates to suppression of melatonin by exposure to light. Neurosci Lett. 2008;440(1): 23–26. doi: 10.1016/j.neulet.2008.05.037 18539392 10.1016/j.neulet.2008.05.037

[63] Ho Mien I, Chua EC, Lau P, Tan LC, Lee IT, Yeo SC, et al Effects of exposure to intermittent versus continuous red light on human circadian rhythms, melatonin suppression, and pupillary constriction. PLoS One. 2014;9(5): e96532 doi: 10.1371/journal.pone.0096532 24797245 10.1371/journal.pone.0096532 PMC4010506

[64] Gooley JJ, Lu J, Fischer D, Saper CB. A broad role for melanopsin in nonvisual photoreception. J Neurosci. 2003;23(18): 7093–7106. 12904470 10.1523/JNEUROSCI.23-18-07093.2003 PMC6740653

[65] Xu Y, Toh KL, Jones CR, Shin JY, Fu YH, Ptacek LJ. Modeling of a human circadian mutation yields insights into clock regulation by PER2. Cell. 2007;128(1): 59–70. doi: 10.1016/j.cell.2006.11.043 17218255 10.1016/j.cell.2006.11.043 PMC1828903

[66] Satoh K, Mishima K, Inoue Y, Ebisawa T, Shimizu T. Two pedigrees of familial advanced sleep phase syndrome in Japan. Sleep. 2003;26(4): 416–417. 12841366 10.1093/sleep/26.4.416

[67] Carpen JD, Archer SN, Skene DJ, Smits M, von Schantz M. A single-nucleotide polymorphism in the 5'-untranslated region of the *hPER2* gene is associated with diurnal preference. J Sleep Res. 2005;14(3): 293–297. doi: 10.1111/j.1365-2869.2005.00471.x 16120104 10.1111/j.1365-2869.2005.00471.x

[68] Matsuo M, Shiino Y, Yamada N, Ozeki Y, Okawa M. A novel SNP in *hPer2* associates with diurnal preference in a healthy population. Sleep Biol Rhythm. 2007;5(2): 141–145. doi: 10.1111/j.1479-8425.2007.00264.x

[69] Lee HJ, Kim L, Kang SG, Yoon HK, Choi JE, Park YM, et al *PER2* variation is associated with diurnal preference in a Korean young population. Behav Genet. 2011;41(2): 273–277. doi: 10.1007/s10519-010-9396-3 20931356 10.1007/s10519-010-9396-3 PMC3044841

[70] Ojeda DA, Perea CS, Niño CL, Gutiérrez RM, López-León S, Arboleda H, et al A novel association of two non-synonymous polymorphisms in *PER2* and *PER3* genes with specific diurnal preference subscales. Neurosci Lett. 2013;553: 52–56. doi: 10.1016/j.neulet.2013.08.016 23969301 10.1016/j.neulet.2013.08.016

[71] Song HM, Cho CH, Lee HJ, Moon JH, Kang SG, Yoon HK, et al Association of *CLOCK*, *ARNTL*, *PER2*, and *GNB3* polymorphisms with diurnal preference in a Korean population. Chronobiol Int. 2016;33(10): 1455–1463. doi: 10.1080/07420528.2016.1231199 27660894 10.1080/07420528.2016.1231199

[72] Hu Y, Shmygelska A, Tran D, Eriksson N, Tung JY, Hinds DA. GWAS of 89,283 individuals identifies genetic variants associated with self-reporting of being a morning person. Nat Commun. 2016;7: 10448 doi: 10.1038/ncomms10448 26835600 10.1038/ncomms10448 PMC4740817

[73] Chellappa SL, Viola AU, Schmidt C, Bachmann V, Gabel V, Maire M, et al Human melatonin and alerting response to blue-enriched light depend on a polymorphism in the clock gene *PER3*. J Clin Endocrinol Metab. 2012;97(3): E433–437. doi: 10.1210/jc.2011-2391 22188742 10.1210/jc.2011-2391

[74] Albrecht U, Zheng B, Larkin D, Sun ZS, Lee CC. *mPer1* and *mPer2* are essential for normal resetting of the circadian clock. J Biol Rhythms. 2001;16(2): 100–104. doi: 10.1177/074873001129001791 11302552 10.1177/074873001129001791

[75] Tamanini F, Yagita K, Okamura H, van der Horst GT. Nucleocytoplasmic shuttling of clock proteins. Methods Enzymol. 2005;393: 418–435. doi: 10.1016/S0076-6879(05)93020-6 15817303 10.1016/S0076-6879(05)93020-6

[76] Okamura H, Miyake S, Sumi Y, Yamaguchi S, Yasui A, Muijtjens M, et al Photic induction of *mPer1* and *mPer2* in *Cry*-deficient mice lacking a biological clock. Science. 1999;286(5449): 2531–2534. doi: 10.1126/science.286.5449.2531 10617474 10.1126/science.286.5449.2531

[77] Shearman LP, Jin X, Lee C, Reppert SM, Weaver DR. Targeted disruption of the *mPer3* gene: subtle effects on circadian clock function. Mol Cell Biol. 2000;20(17): 6269–6275. doi: 10.1128/MCB.20.17.6269–6275.2000 10938103 10.1128/mcb.20.17.6269-6275.2000 PMC86101

[78] Hirayama J, Sassone-Corsi P. Structural and functional features of transcription factors controlling the circadian clock. Curr Opin Genet Dev. 2005;15(5): 548–556. doi: 10.1016/j.gde.2005.07.003 16095901 10.1016/j.gde.2005.07.003

[79] Hennig S, Strauss HM, Vanselow K, Yildiz O, Schulze S, Arens J, et al Structural and functional analyses of PAS domain interactions of the clock proteins *Drosophila* PERIOD and mouse PERIOD2. PLoS Biol. 2009;7(4): e94 doi: 10.1371/journal.pbio.1000094 19402751 10.1371/journal.pbio.1000094 PMC2671562

[80] Oota H, Pakstis AJ, Bonne-Tamir B, Goldman D, Grigorenko E, Kajuna SLB, et al The evolution and population genetics of the *ALDH2* locus: random genetic drift, selection, and low levels of recombination. Ann Hum Genet. 2004;68(2): 93–109. doi: 10.1046/j.1529-8817.2003.00060.x 15008789 10.1046/j.1529-8817.2003.00060.x

[81] Han Y, Gu S, Oota H, Osier MV, Pakstis AJ, Speed WC, et al Evidence of positive selection on a class I *ADH* locus. Am J Hum Genet. 2007;80(3): 441–456. doi: 10.1086/512485 17273965 10.1086/512485 PMC1821113

[82] Nakagome S, Mano S, Kozlowski L, Bujnicki JM, Shibata H, Fukumaki Y, et al Crohn's disease risk alleles on the *NOD2* locus have been maintained by natural selection on standing variation. Mol Biol Evol. 2012;29(6): 1569–1585. doi: 10.1093/molbev/mss006 22319155 10.1093/molbev/mss006 PMC3697811

[83] Tishkoff SA, Williams SM. Genetic analysis of African populations: human evolution and complex disease. Nat Rev Genet. 2002;3(8): 611–621. doi: 10.1038/nrg865 12154384 10.1038/nrg865

[84] Kidd KK, Pakstis AJ, Speed WC, Kidd JR. Understanding human DNA sequence variation. J Hered. 2004;95(5): 406–420. doi: 10.1093/jhered/esh060 15388768 10.1093/jhered/esh060

