# Supplementary figures and images for: An ancestral haplotype of the human PERIOD2 gene associates with reduced sensitivity to light-induced melatonin suppression
Source: PLoS One. 2017 Jun 26;12(6):e0178373. doi: 10.1371/journal.pone.0178373 (PMC5484468; doi:10.1371/journal.pone.0178373)

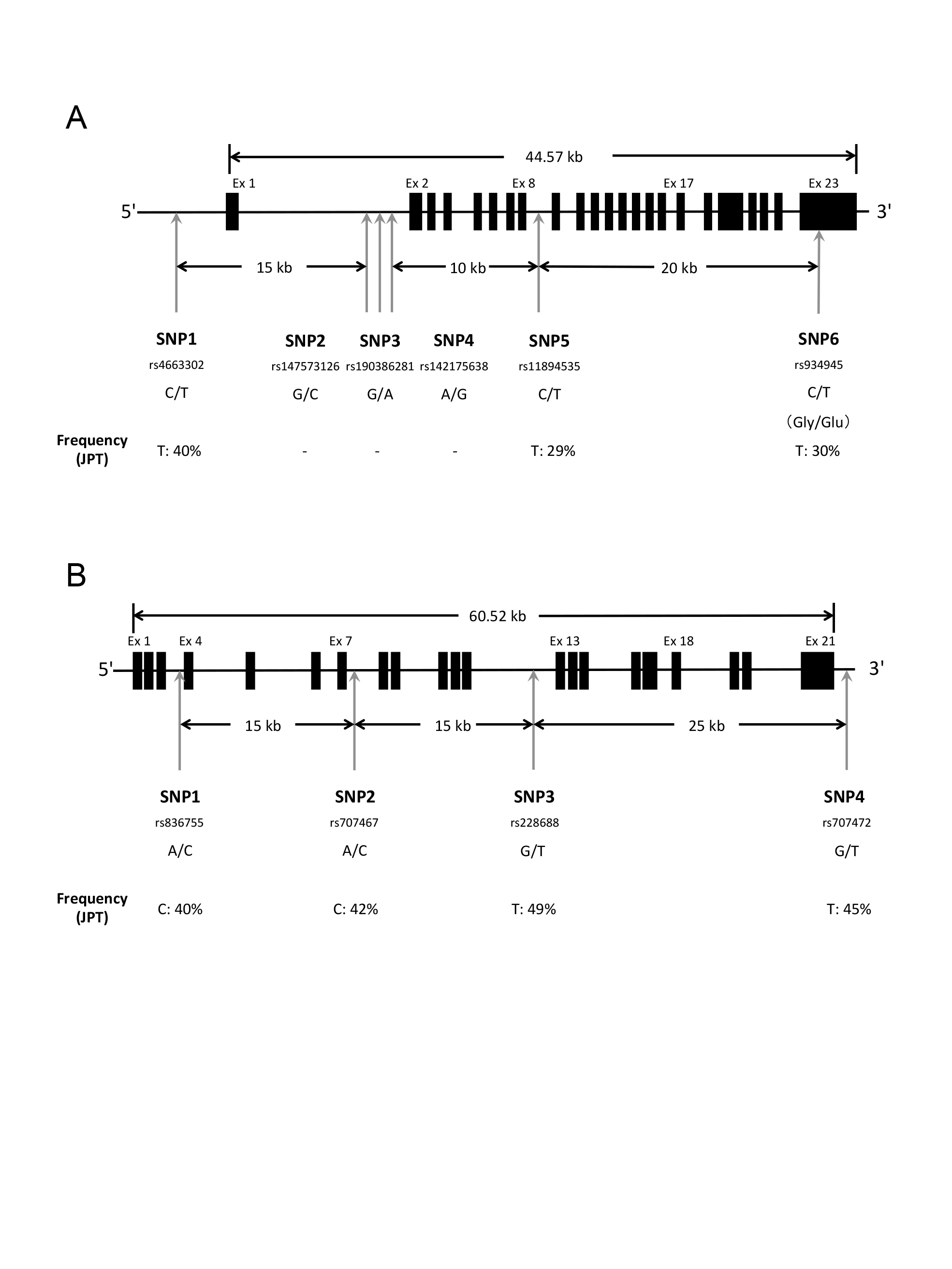

Supplement: S1 Fig — (A) Six SNPs on PER2 and (B) four SNPs on PER3 are numbered serially, and their rs numbers, alleles reported, and allele frequencies of JPT in the HapMap database are shown respectively. (TIF) [file pone.0178373.s001.tif]

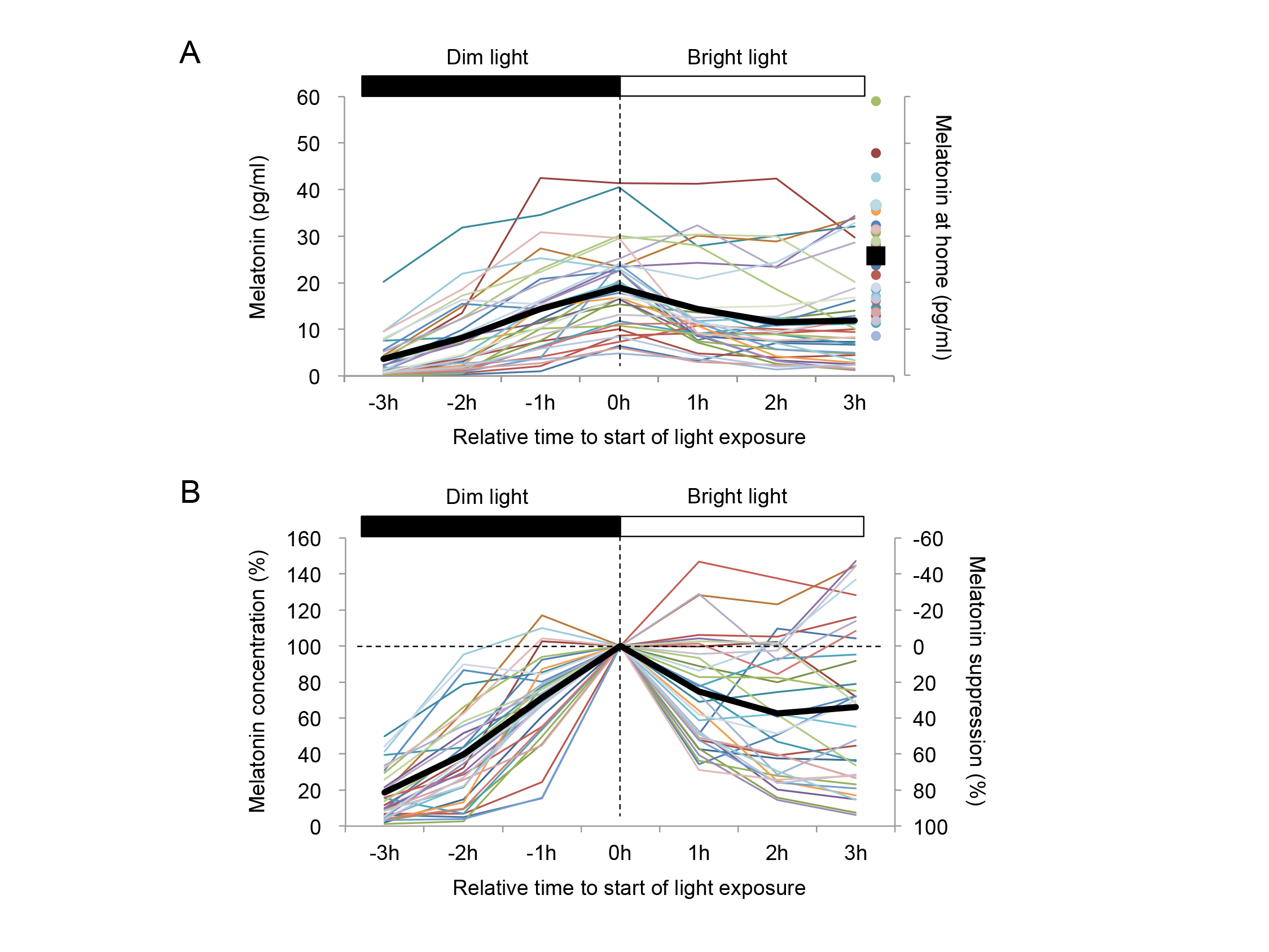

Supplement: S2 Fig — (A) The raw data of melatonin concentration in all the subjects. The bold line shows the average. The start time of light exposure (0 h) was individually set 3 and 3.5 hours before the midpoint of sleep of each subject. There was a large interindividual difference. The dots shows the melatonin concentration measured at home at the same time as 3 hours after light exposure (3 h) 2 days before the experiment. (B) The percentage of melatonin concentration (left vertical axis) and the percentage of melatonin suppression by light (right vertical axis), which were calculated based on the data before light exposure (0 h). Although melatonin concentration increased during dim light in all the subjects, there was a large interindividual difference in the percentage of melatonin suppression. (TIF) [file pone.0178373.s002.tif]

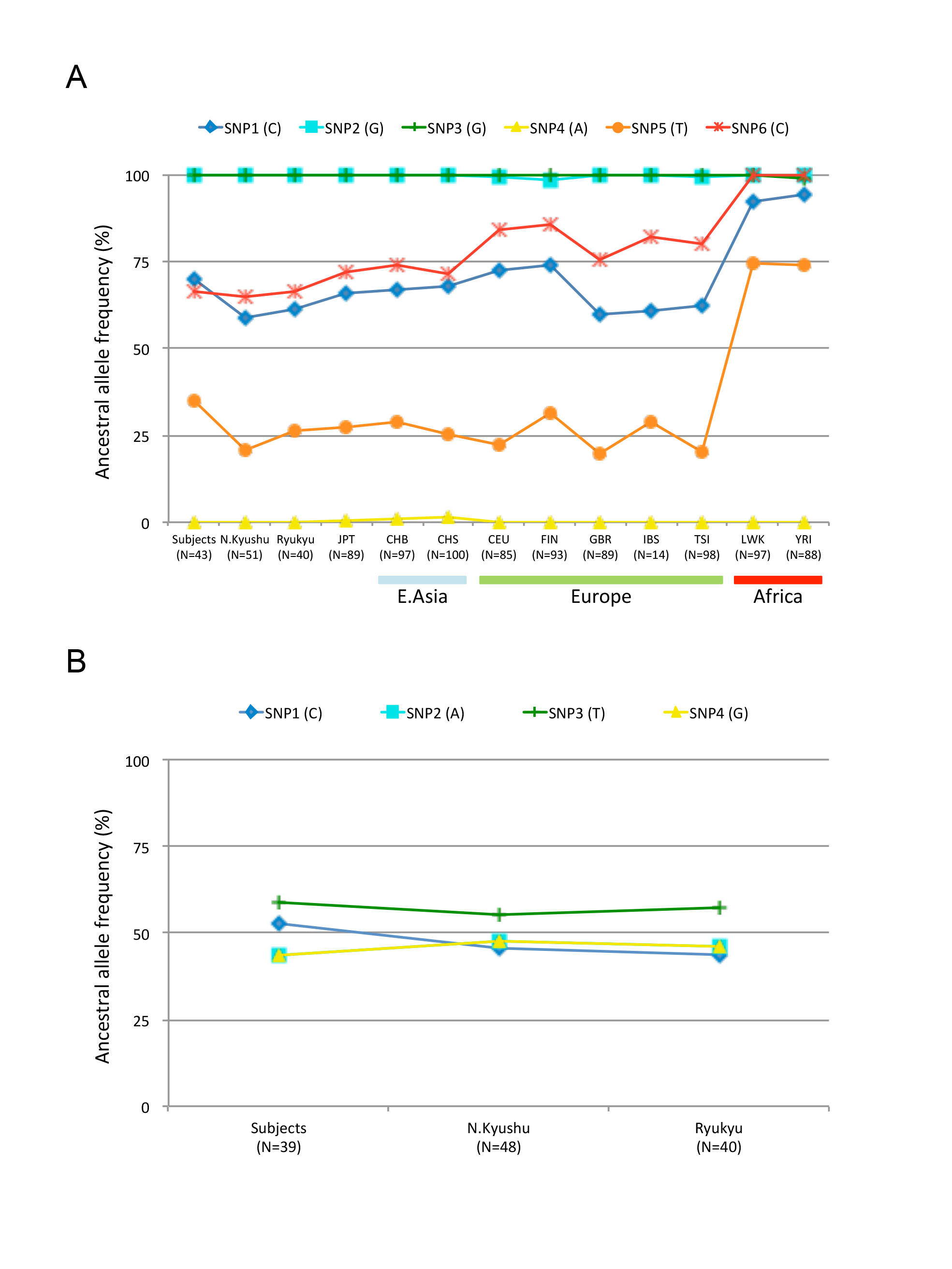

Supplement: S3 Fig — Ancestral allele frequencies of SNPs (A) in PER2 and (B) in PER3. Three groups, Subjects, Northern Kyushu and Ryukyu were genotyped in this study. N represents the number of individuals. (TIF) [file pone.0178373.s003.tif]

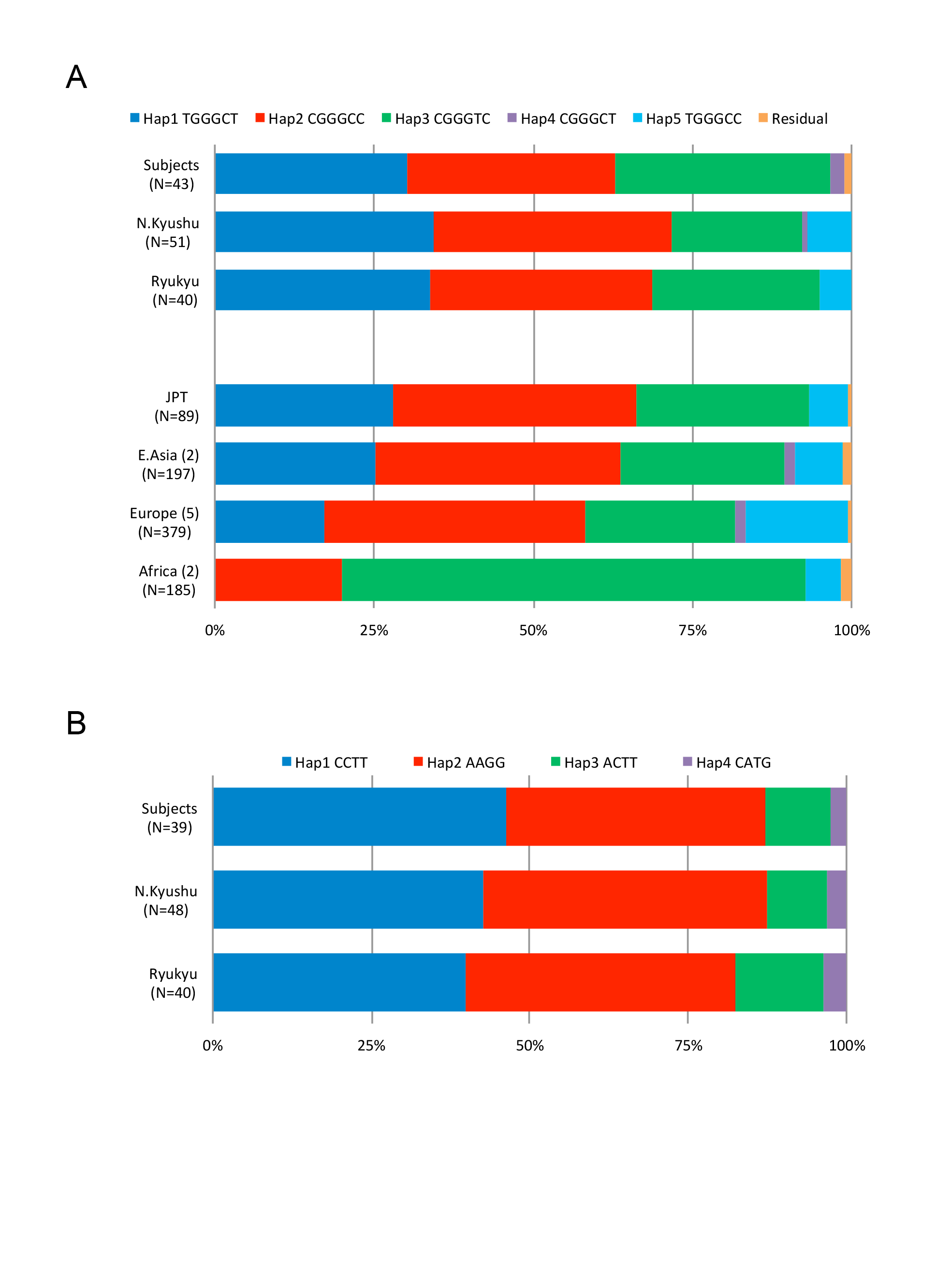

Supplement: S4 Fig — Haplotype frequencies for (A) PER2 and (B) PER3. The numbers of local populations included in the geographical region are shown in parentheses. N represents the numbers of the individuals. The combined frequencies of the remaining haplotype (Residuals) are less than 1.0% in all the geographical regions. (TIF) [file pone.0178373.s004.tif]

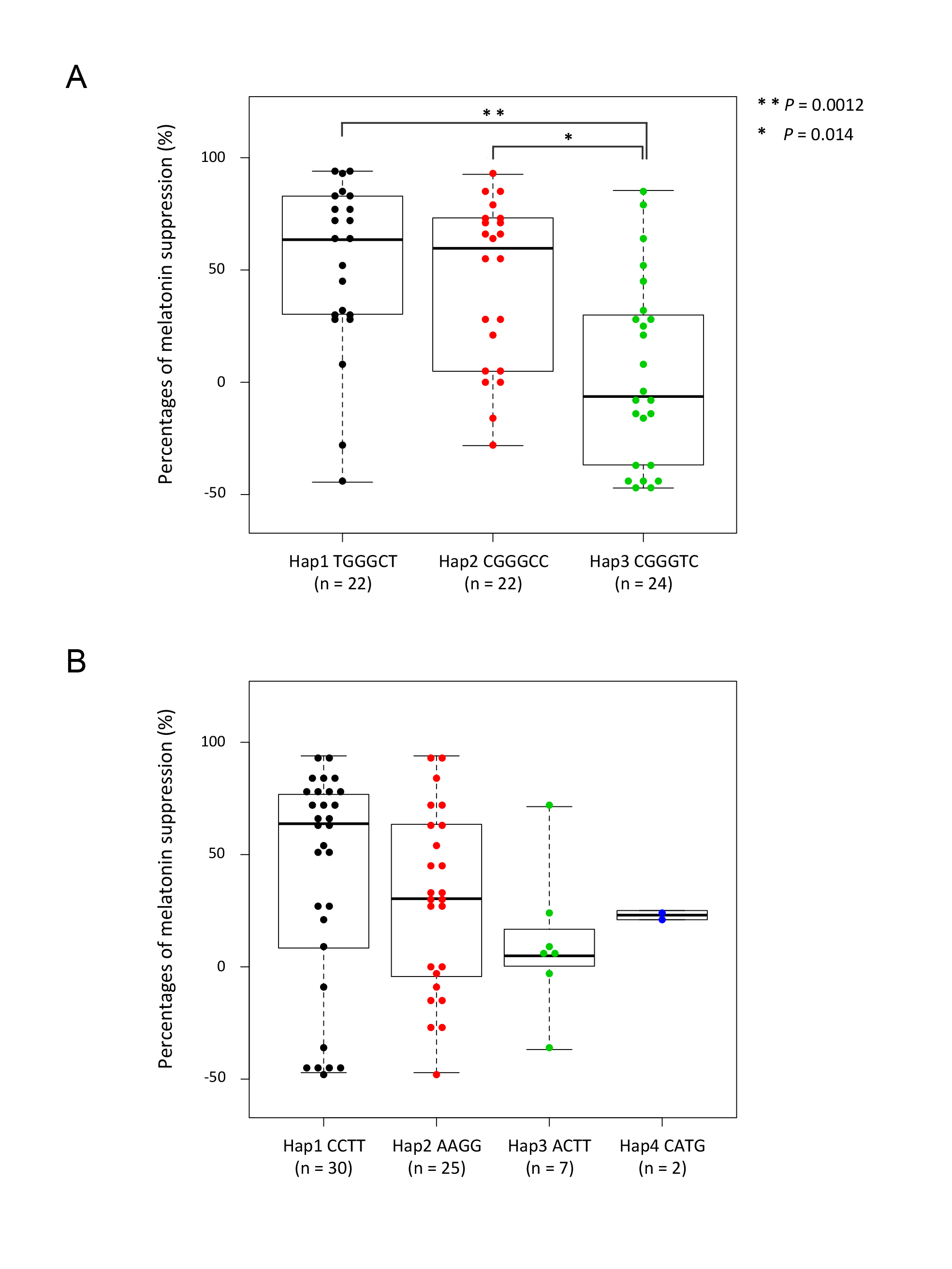

Supplement: S5 Fig — Comparison of the distributions of percentages of melatonin suppression for major haplotypes of (A) PER2 and (B) PER3. The thick middle lines in the boxes represent the medians, and the tops and bottoms of the boxes represent the third and the first quartiles, respectively. One dot represents one chromosome, and the numbers of chromosomes, n, are shown in parentheses. (TIF) [file pone.0178373.s005.tif]

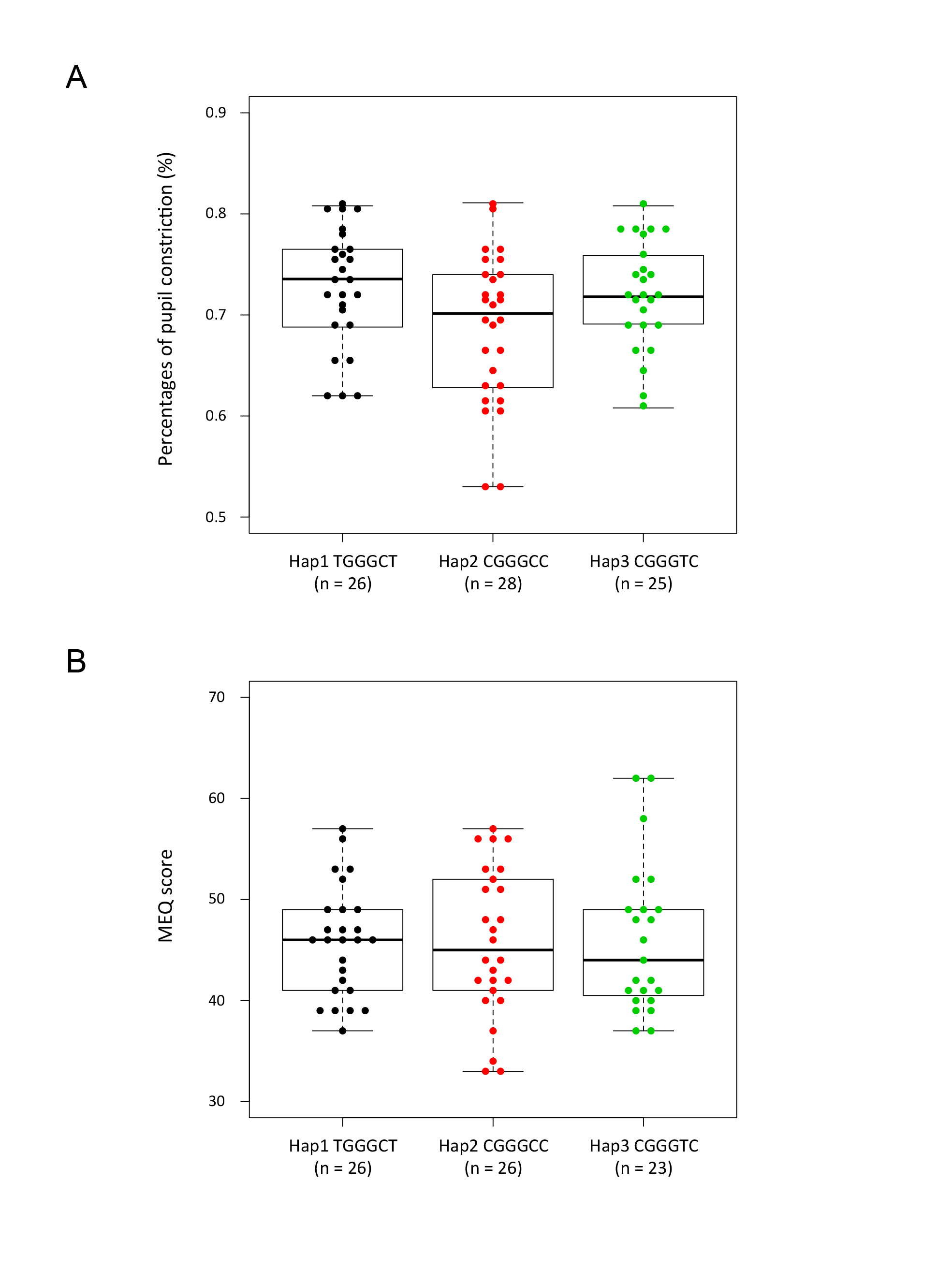

Supplement: S6 Fig — Comparison of the distributions of (A) percentages of pupil constriction and (B) MEQ score for three major haplotypes of PER2. Thick middle lines in the boxes represent the medians, and the tops and bottoms of the boxes represent the third and the first quartiles, respectively. One dot represents one chromosome, and the numbers of chromosomes, n, are shown in parentheses. (TIF) [file pone.0178373.s006.tif]

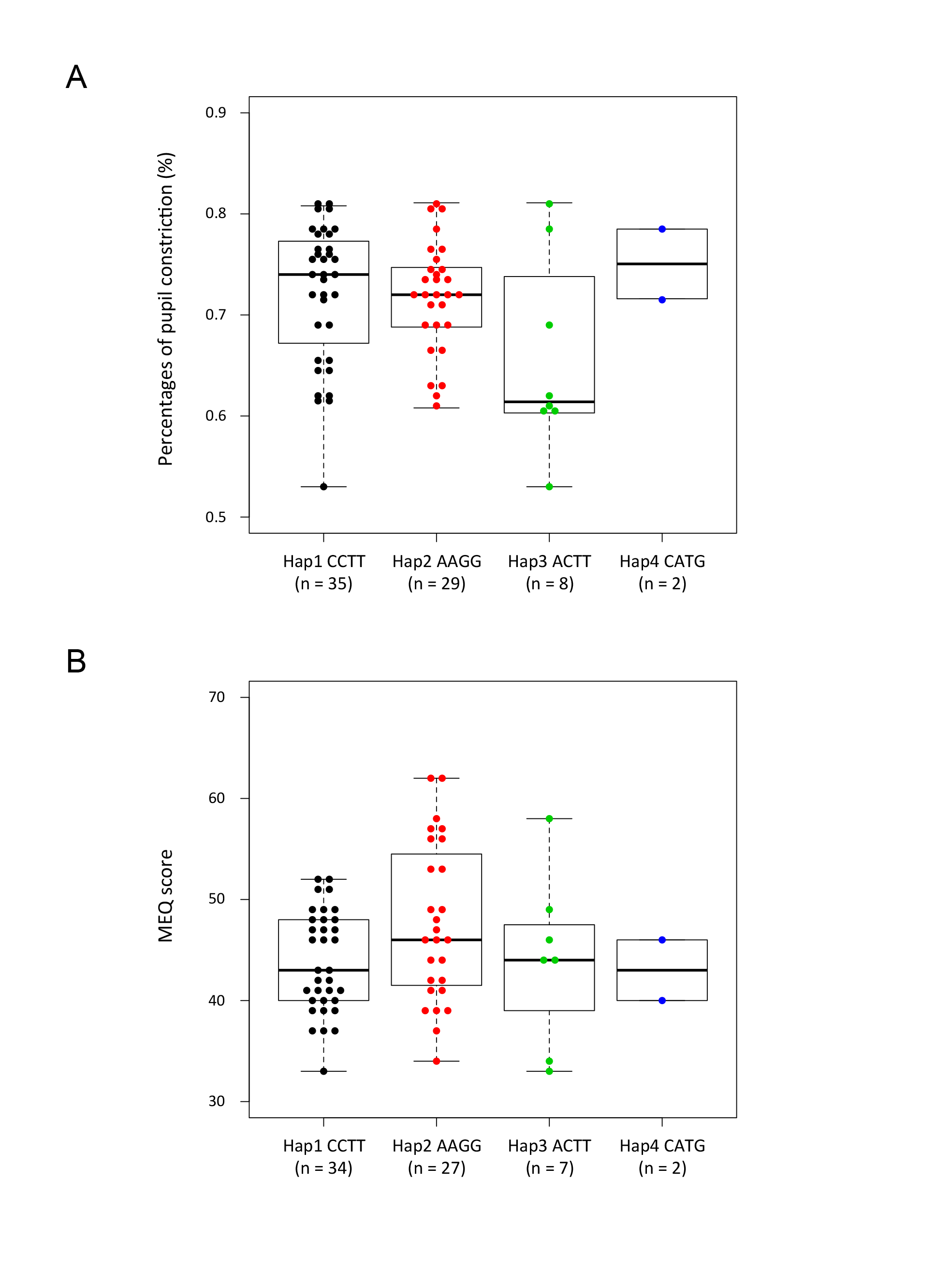

Supplement: S7 Fig — Comparison of the distributions of (A) percentages of pupil constriction and (B) MEQ score for four major haplotypes of PER3. The thick middle lines in the boxes represent the medians, and the tops and bottoms of the boxes represent the third and the first quartiles, respectively. One dot represents one chromosome, and the numbers of chromosomes, n, are shown in parentheses. (TIF) [file pone.0178373.s007.tif]

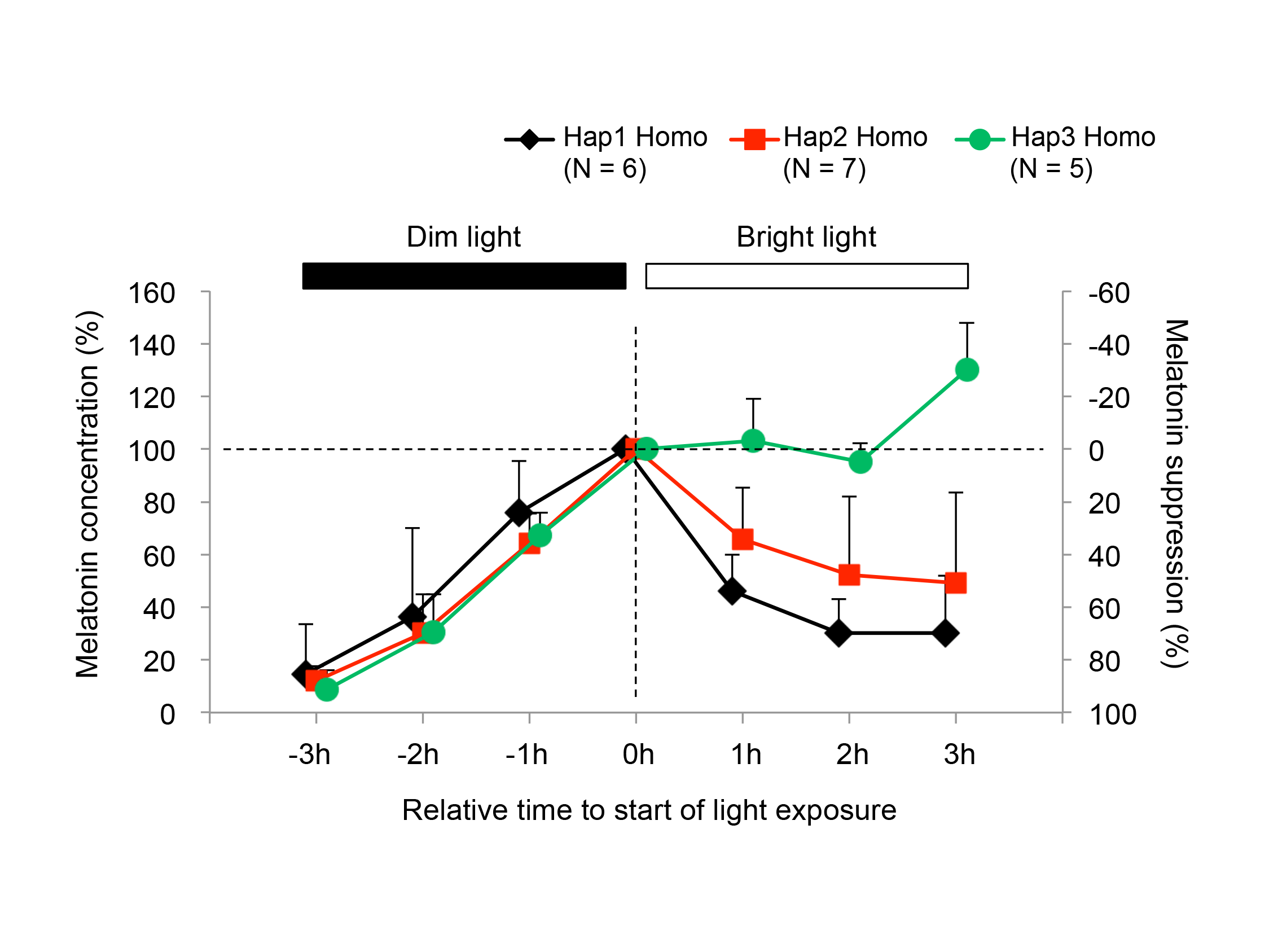

Supplement: S8 Fig — The percentages of melatonin suppression by light exposure in Hap1, Hap2, and Hap3 homozygotes. The data are shown as the mean + SD. (TIF) [file pone.0178373.s008.tif]

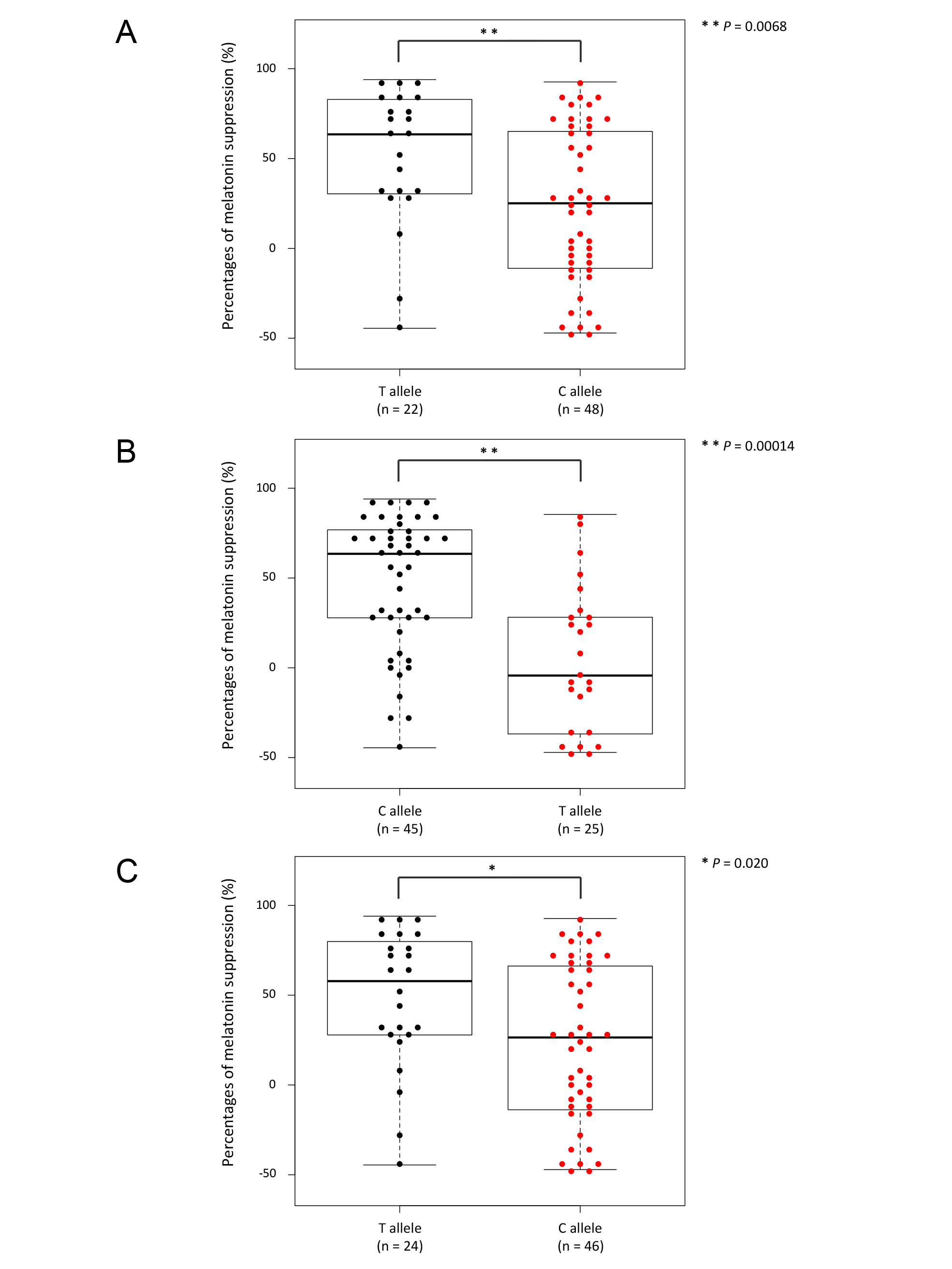

Supplement: S9 Fig — Comparison of the distributions of percentages of melatonin suppression for PER2 alleles of (A) SNP1, (B) SNP5, and (C) SNP6. The thick middle lines in the boxes represent the medians, and the tops and bottoms of the boxes represent the third and the first quartiles, respectively. One dot represents one chromosome, and the numbers of chromosomes, n, are shown in parentheses. The Kruskal-Wallis test shows statistically significant differences. (TIF) [file pone.0178373.s009.tif]

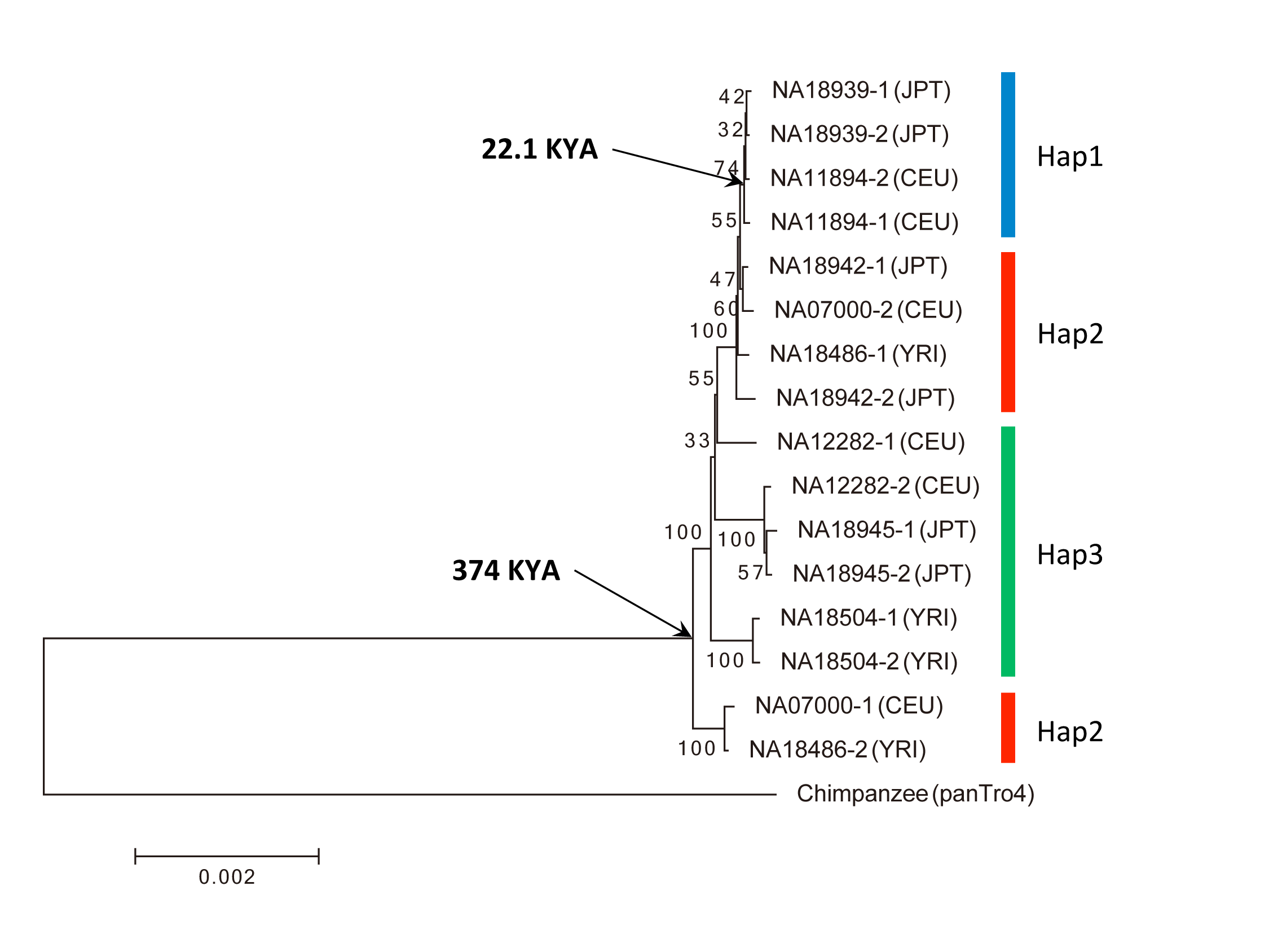

Supplement: S10 Fig — The phased sequences that had homozygotes of three major haplotypes (Hap1, Hap2, and Hap3) in YRI, CEU, and JPT were obtained from the 1000 Genome Project (phase 3) database. The arrows represent divergence times. (TIF) [file pone.0178373.s010.tif]
